# Rhizoma Gastrodiae Water Extract Modulates the Gut Microbiota and Pathological Changes of P-Tau^Thr231^ to Protect Against Cognitive Impairment in Mice

**DOI:** 10.3389/fphar.2022.903659

**Published:** 2022-07-15

**Authors:** Wenbin Zhao, Jianhui Wang, Maria Latta, Chenyu Wang, Yuheng Liu, Wantong Ma, Zhongkun Zhou, Shujian Hu, Peng Chen, Yingqian Liu

**Affiliations:** ^1^ School of Pharmacy, Lanzhou University, Lanzhou, China; ^2^ Department of Pathology, Yale University School of Medicine, New Haven, CT, United States; ^3^ School of Pharmacy, University of Connecticut, Mansfield, CT, United States; ^4^ School of Basic Medical Science, Lanzhou University, Lanzhou, China

**Keywords:** Rhizoma Gastrodiae water extract, Alzheimer’s disease, P-Tau protein, gut microbiota, *Lactobacillus johnsonii*, *Lactobacillus murinus*, *Lactobacillus reuteri*

## Abstract

Gastrodiae Rhizoma and its active constituents are known to exhibit neuroprotective effects in Alzheimer’s disease (AD). However, the effect of Rhizoma Gastrodiae water extract (WERG) on AD and the detailed mechanism of action remain unclear. In this study, the mechanism of action of WERG was investigated by the microbiome–gut–brain axis using a D-galactose (D-gal)/AlCl_3_-induced AD mouse model. WERG improved the cognitive impairment of D-gal/AlCl_3_-induced mice. The expression level of p-Tau^thr231^ in the WERG-H treatment group was decreased, and p-Tau^thr231^ was found negative in hippocampal DG, CA1, and CA3 regions. Here, the diversity and composition of the gut microbiota were analyzed by 16sRNA sequencing. WERG-H treatment had a positive correlation with Firmicutes, Bacilli, *Lactobacillus johnsonii*, *Lactobacillus murinus*, and *Lactobacillus reuteri*. Interestingly, the Rikenellaceae-RC9 gut group in the gut increased in D-gal/AlCl_3_-induced mice, but the increased *L. johnsonii*, *L. murinus*, and *L. reuteri* reversed this process. This may be a potential mechanistic link between gut microbiota dysbiosis and P-Tau^Thr231^ levels in AD progression. In conclusion, this study demonstrated that WERG improved the cognitive impairment of the AD mouse model by enriching gut probiotics and reducing P-Tau^Thr231^ levels.

## Introduction

Along with the rapid increase in the elderly population (≥ 65 years) worldwide, Alzheimer’s disease (AD) is now considered a major global public health threat and causes a huge economic burden (Prince et al., 2015; Li et al., 2021). AD, a primary degenerative brain disease, is caused by synaptic lesions and neuronal loss (An et al., 2017). The disease is clinically characterized by amyloid plaques and neurofibrillary tangles (NFT), as well as progressive cognitive impairment and memory loss (Prakash and Kumar, 2014). Intraneuronal accumulation of hyperphosphorylated tau is a hallmark pathology shown in over 20 neurodegenerative disorders, which is collectively termed tauopathies, including AD (Zheng et al., 2021). Numerous studies have shown that abnormal hyperphosphorylated tau protein plays an important role in the occurrence and development of neurodegeneration and learning, and memory impairment in AD (Sahara et al., 2010). Therefore, selectively removing or reducing hyperphosphorylated tau is promising for therapies of AD and other tauopathies. However, due to the complex etiology and pathogenesis, there is currently no strategy for specifically targeting tau phosphorylation.

To date, the traditional Chinese herbal medicine has several thousand years’ history as a drug for AD in oriental countries (Chang et al., 2015). Rhizoma Gastrodiae is a perennial parasitic herbal with neuroprotective activities (Zhan et al., 2016) and has shown that it has positive effects on the central nervous system, cardiovascular system, and immune system (Lee et al., 2012; Shu et al., 2013). Some components from Rhizoma Gastrodiae, such as gastrodin, have been reported to suppress inflammation and attenuate liver injury by modulating gut microbiota (Liu et al., 2021a; Ma et al., 2021). Homogeneous polysaccharide GEP-1 can promote the growth of *Akkermansia muciniphila* (*A. muciniphila*) and *Lacticaseibacillus paracasei* (*L. paracasei*) strains (Huo et al., 2021). Rhizoma Gastrodiae water extract (WERG) modulates neurotransmitters and alters the gut microbiota in a depression mouse model (Huang et al., 2021). Multiple probiotics grew after taking fresh Rhizoma Gastrodiae extract, including *Ruminiclostridium*, *Butyricicoccus*, and *Parvibacter* (Hua et al., 2019). It produced a positive regulation on the mouse gut microbiota (Hua et al., 2019). However, the anti-Alzheimer’s effects of WERG in AD mouse models were little studied. This study aimed to explore the health-promoting effects of long-term WERG intervention on the AD mouse model.

Gut microbiota, also known as “the second brain,” can regulate brain function (Hsiao et al., 2013). Growing evidence suggested that there is an association between the gut–brain axis and AD progression. The gut microbiota affects the brain and behavior through pathways such as the vagus nerve, microbial metabolites, immune stimulation, enteroendocrine cells, the enteric nervous system, and neurotransmitters (Nagpal and Cryan, 2021). Gut microbiota can promote AD pathology, cognitive impairment, and microglial activation in AD mice (Chen et al., 2022). Meanwhile, gut microbiota affects various complex behaviors, including emotional, social, and anxiety-like behaviors (Hsiao et al., 2013). Previous studies reported that the traditional Chinese medicine prescription “Huanglian Jiedu Decoction” could reverse the cognitive impairment of Tg mice, reshape the gut microbiota structure of Tg mice, and enrich the population of short-chain fatty acid-producing gut microbiota (Gu et al., 2021). GV-971 is a new drug for Alzheimer's disease originally developed in China and the first in the world targeting the brain–gut axis. Studies have shown that it can significantly improve the memory dysfunction of animals caused by tau phosphorylation, Aβ deposition, and neuroinflammation (Rao, 2020). Mannan oligosaccharides alleviated cognitive and behavioral impairments in 5xFAD Alzheimer’s mice by modulating the gut–microbiota–brain axis (Liu et al., 2021b). However, the role of gut microbiota in AD pathogenesis remains unclear. D-Galactose/aluminum chloride (D-gal/AlCl_3_) can induce AD-like symptoms (Zhang et al., 2016a; Wei et al., 2017). Animals exposed to long-term D-gal show aging-related changes such as elevated oxidant levels and cognitive impairment (Lei et al., 2011; Yang et al., 2013). Furthermore, intraperitoneal injection (i.p) of D-gal resulted in increased levels of acetylcholinesterase in the brains of rats (Gao et al., 2016). Aluminum has been linked to the pathogenesis of AD (Kaizer et al., 2008). Accumulating evidence showed that co-administration of D-gal/AlCl_3_ to rats impaired their cognitive functions, increased AChE activities, altered oxidative balance, and induced neurodegeneration (Zhang et al., 2016b; Li et al., 2016; Chiroma et al., 2018). As a consequence, this study evaluated the health-promoting effects of WERG on D-gal/AlCl_3_-induced mice *via* the microbiome–gut–brain axis, and provides a theoretical basis and a new perspective for the development and utilization of WERG.

## Materials and Methods

### Chemicals and Materials

Rhizoma Gastrodiae comes from Yangba, Kang County, Gansu Province, China. Yangba Town is located 84 km southeast of Kang County. Accurately 10.0 g of Gastrodia elata was weighed, the appropriate amount of distilled water soaked for 1 h was added and boiled three times (each time for 30 min), frying was stopped and the mixture was cooled to room temperature, gauze was filtered, the filtrate was combined, and the filtrate was concentrated to 1.0 ml of crude drug per ml of distilled water decoction. Oxiracetam (99%) was purchased from McLean Biochemical Technology Co., Ltd., Shanghai, China.

### Animals

All mice were housed 10 per cage and maintained under 12 h light–dark cycle, temperature (23 ± 1°C), humidity (60% ± 10%), and SPF conditions with free access to food and water. The protocol was approved by the guidelines of the Lanzhou Institute of Animal Science.

### Experimental Design and Drug Treatment in D-gal and AlCl_3_-Induced Mice

**FIGURE 1:**
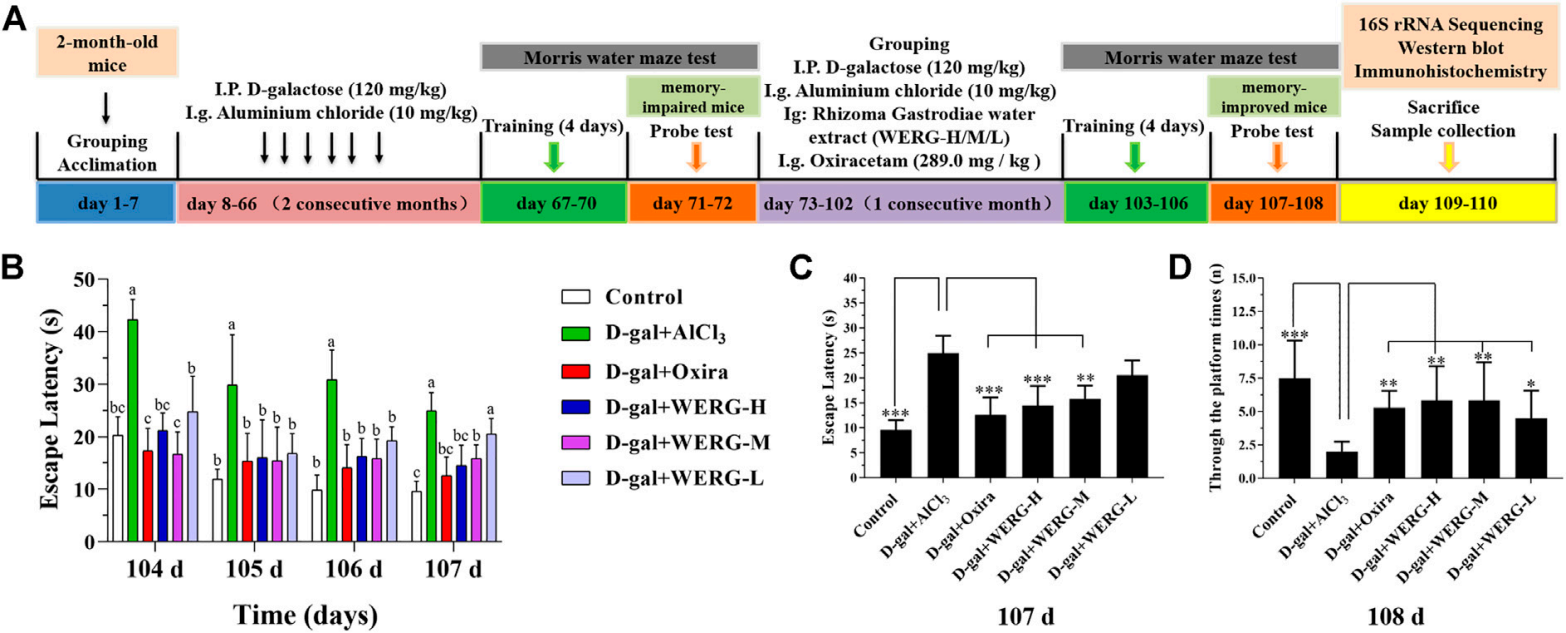
WERG-H improved cognitive impairment in AD mouse model. Control, D-gal 120 + AlCl_3_ 10 mg/kg. bwt (D-gal + AlCl_3_), D-gal 120 + AlCl_3_ 10 + Oxiracetam 298 mg/kg. bwt (D-gal + Oxira), D-gal 120 + AlCl_3_ 10 + Rhizoma Gastrodiae water extract 300 mg/kg. bwt (D-gal + WERG-H), D-gal 120 + AlCl_3_ 10 + Rhizoma Gastrodiae water extract 200 mg/kg. bwt (D-gal + WERG-M), D-gal 120 + AlCl_3_ 10 + Rhizoma Gastrodiae water extract 100 mg/kg. bwt (D-gal + WERG-L). Experimental protocols and prevention strategies **(A)**; the escape latency during training (104–107 days) **(B)**; escape latency (107 days) **(C)**; after removal of the platform, through the platform times (90 s) **(D)**. Data were expressed as mean ± SD (*n* = 8). “*” presented significant difference at *p* < 0.05 levels, “**” presented significant difference at *p* < 0.01 level, “***” presented significant difference at *p* < 0.001 level.

SPF-grade two-month-old mice were divided into six groups with eight mice in each group, the first two months: 1) control group (distilled water + physiological saline solution); (2–6) treated groups (120 mg/kg D-galactose + 10 mg/kg AlCl_3_ daily); three-month: 1) control group (distilled water + physiological saline solution daily); 2) D-gal + AlCl_3_ group (120 mg/kg D-galactose + 10 mg/kg AlCl_3_ daily); 3) D-gal + Oxira group (120 mg/kg D-galactose + 10 mg/kg AlCl_3_ + 289.0 mg/kg oxiracetam daily); 4) D-gal + WERG-L group (120 mg/kg D-galactose + 10 mg/kg AlCl_3_ + 100 mg/kg WERG daily); 5) D-gal + WERG-M group (120 mg/kg D-galactose + 10 mg/kg AlCl_3_ + 200 mg/kg WERG daily); 6) D-gal + WERG-H group (120 mg/kg D-galactose + 10 mg/kg AlCl_3_ + 300 mg/kg WERG daily). Oxiracetam was chosen as a positive control. Figure 1A showed the experimental design and drug treatment schedule.

### Morris Water Maze Test

MWMT conditions are as follows: diameter 1.5 m, water depth 21 cm, platform diameter 8 cm, height 20 cm, water temperature 25°C, and milky white water. Navigation test: Mice were randomly placed into the pool and let to swim for 60 s to find the hidden platform. The time required for the mouse to climb on the platform was used as escape latency and stayed for 15 s. If the platform could not be found within 60 s, the escape latency was recorded as 60 s, and it was placed on the platform for 15 s. According to this method, each animal was trained twice a day for four consecutive days. Probe test: After the platform was removed, mice were randomly placed into the water for 90 s, and the number of original platform crossings was recorded.

### Western Blot

Western blots were carried out as the previously described method (Cui et al., 2019) with some modifications. Total proteins were extracted from hippocampus tissues using RIPA lysis buffer (MCE, Shanghai, China). The primary antibody was purchased from Bioss (Bioss, Beijing, China, 1:500). Goat-anti-rabbit IgG secondary antibody was used as the secondary antibody (Bioss, Beijing, China, 1:3000). The grayscale analysis of Western blot results was evaluated by ImageJ software.

### Hematoxylin and Eosin Staining and Immunohistochemical Staining

Immunohistochemistry was carried out as the previously described method (Zhang et al., 2020) with some modifications. For immunohistochemical staining, the sections were incubated with P-tau (Thr231) antibody (Bioss, Beijing, China, 1:300) and GFAP antibody (Bioss, Beijing, China, 1:200) overnight at 4°C. After washing, the sections were incubated with the appropriate secondary antibody (Bioss, Beijing, China) for 60 min at 25°C. Finally, these sections were observed by using a fluorescence digital photo microscope (OLYMPUS, Japan).

### 16 S rRNA Sequencing

The genomic DNA of feces was extracted using the CTAB/SDS method, and then the purity and concentration of DNA were detected by agarose gel electrophoresis. An appropriate amount of the sample was taken in a centrifuge tube, and the sample was diluted to 1 ng/μl with sterile water. PCR products were detected by electrophoresis on a 2% agarose gel. Equal amounts of samples were mixed according to the concentration of PCR products, 2% agarose gel electrophoresis was used to detect PCR products after mixing thoroughly, and the target bands were recovered. The TruSeq^®^ DNA PCR-Free Sample Preparation Kit was used for library construction. The constructed library was quantified by Qubit and Q-PCR. After the library was qualified, Illumina HiSeq2500 PE250 was used for on-machine sequencing.

### Statistical Analysis

Data are presented as mean ± SEM. The experimental data were analyzed using SPSS version 22.0. A *p*-value < 0.05 was considered to be statistically significant, and Duncan’s statistical procedure was performed.

## Results

### WERG Treatment Ameliorated D-gal/AlCl_3_-Induced Cognitive Impairment and Changes in p-Tau^Thr231^ Protein Expression

**FIGURE 2:**
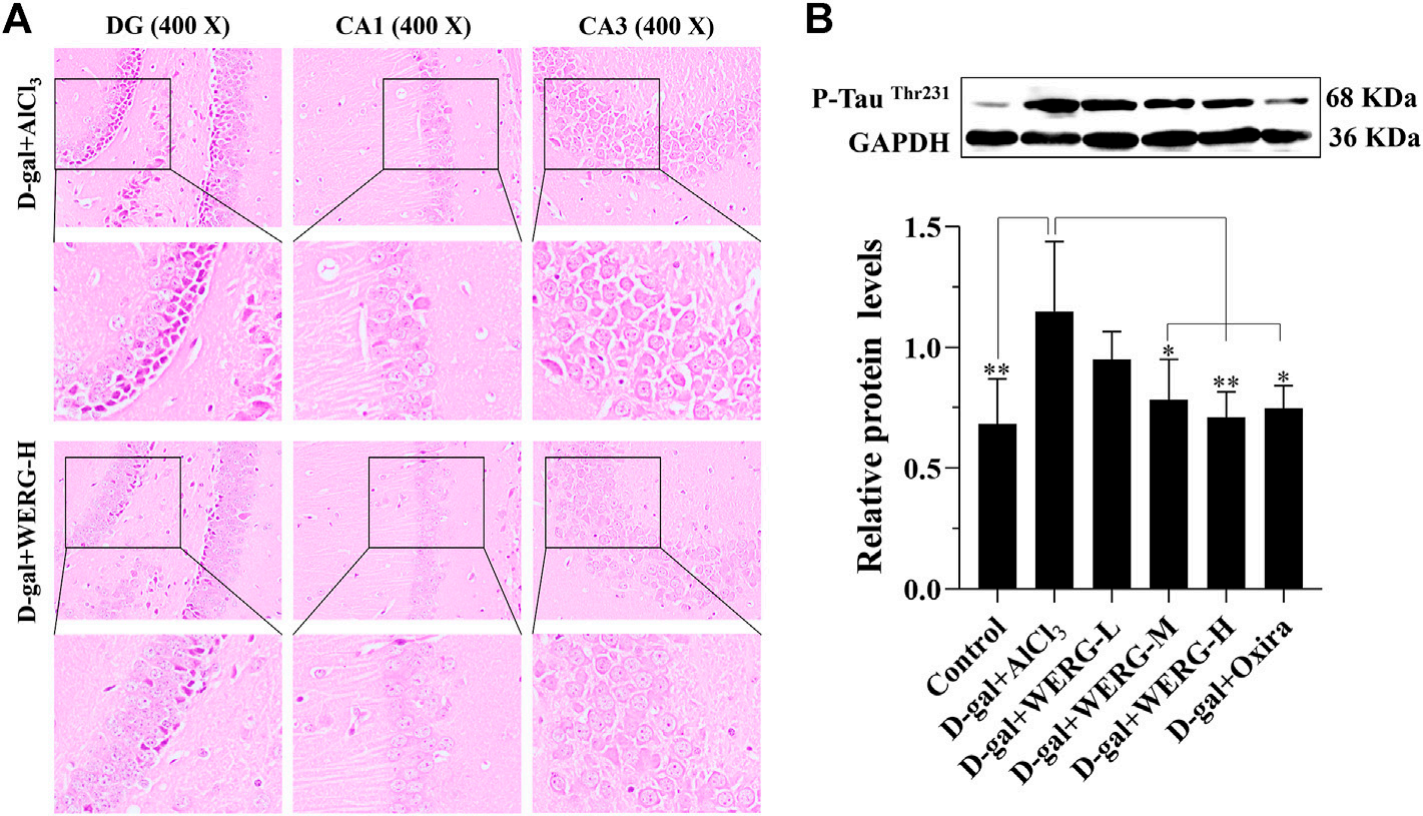
WERG decreased hippocampus neuron damage in an AD mouse model. Pathological changes in the hippocampus CA1, CA3, and DG regions were detected by HE staining (400 ×) **(A)**; the expression of p-TauThr231 was quantitatively analyzed by Western blotting **(B)**. Data were presented as mean ± SD repeated three times. “*” presented significant difference at *p* < 0.05 levels, “**” presented significant difference at *p* < 0.01 level.

**FIGURE 3:**
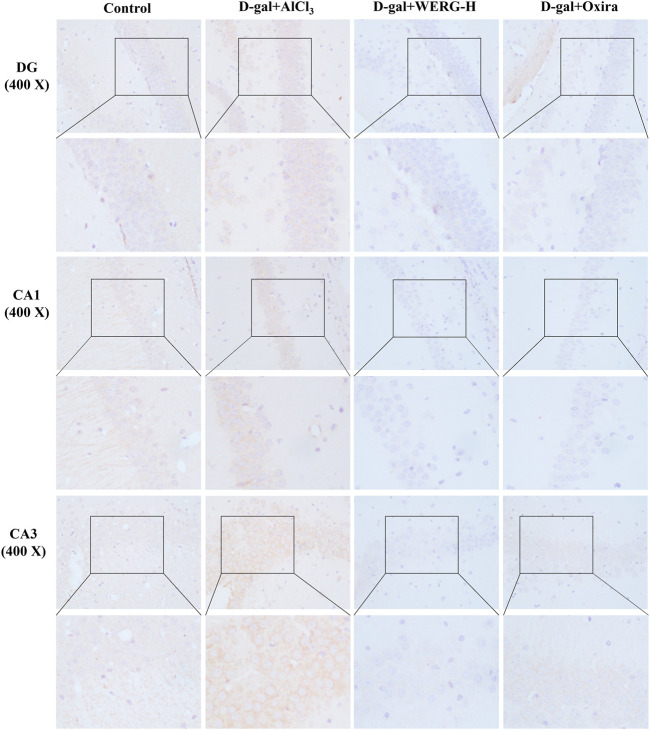
Th1e expressions of p-Tau^Thr231^ were measured by immunohistochemistry. The sections of the DG, CA1, and CA3 regions were acquired using a fluorescence digital photo microscope (OLYMPUS, Japan) at × 400 magnification (scale bar, 100 μm).

To investigate the improving effects of WERG on the D-gal/AlCl_3_-induced mice, two cognition-related indicators were examined in mice. The escape latency of each group was improved in a time-dependent manner during the training process, and the D-gal/AlCl_3_ group was significantly different from the control group, indicating that the AD mouse model induced by D-gal/AlCl_3_ was effective (Figure 1B). After training, the escape latency at 107 days was significantly different between the treated and D-gal/AlCl_3_ groups, especially in D-gal + WERG-H and D-gal + Oxira groups (*p* < 0.001) (Figure 1C). The D-gal + WERG-H and D-gal + Oxira groups also had an increased number of through the platform times compared with the D-gal/AlCl_3_ group (*p* < 0.01) (Figure 1D). As shown in Figure 2A, the results of HE staining showed that a large number of swollen neurons with loosen structure, karyopyknosis, and other morphological changes could be observed in hippocampus neurons of CA3 and DG regions in the D-gal/AlCl_3_ group. When compared with the D-gal/AlCl_3_ group, the pathological changes of hippocampus neurons were ameliorated in the D-gal + WERG-H group, and the tissue cells in the DG and CA3 regions of the hippocampus were generally lighter in staining, with clear cell boundaries and neat arrangement. In addition, CA1 region hippocampus neurons had no obvious pathological changes in the two groups. Western blot analysis indicated that the levels of p-Tau^Thr231^ in the D-gal + WERG-H group were decreased compared with those of the D-gal/AlCl_3_ group (*p* < 0.01); WERG down-regulated the levels of p-Tau^Thr231^ in a dose-dependent manner (Figure 2B). We further investigated the expression levels of p-Tau^Thr231^ by immunohistochemistry, and a semi-quantitative analysis was performed (Figure 3). The distribution pattern of p-Tau^Thr231^ in the CA1, CA3, and DG regions of four treatment groups was examined (Table 1). The number of p-Tau^Thr231^-positive cells in the D-gal + AlCl_3_ group was significantly increased compared with that in the control group, and the arrangement was scattered, the overall staining was darker, and the cytoplasm of the cells was brown. The plaques were significantly increased and darker than those in the WERG-H group. Compared with the D-gal + AlCl_3_ group, the number of positive cells in the WERG-H group decreased, the brown plaques became lighter, and the cells were compactly arranged.

**TABLE 1:** P-Tau^Thr231^-positive structures in D-gal/AlCl_3_-induced AD-related tauopathy.

Hippocampus	D-gal + AlCl_3_			D-gal + Oxira			D-gal + WERG-H	
CA1	−	+	+	−	+	−	−	−
CA3	−	+	+	−	+	+	−	−
DG	−	−	+	−	+	−	−	−

### The Diversity and Richness of Gut Microbiota Was Changed by WERG

**FIGURE 4:**
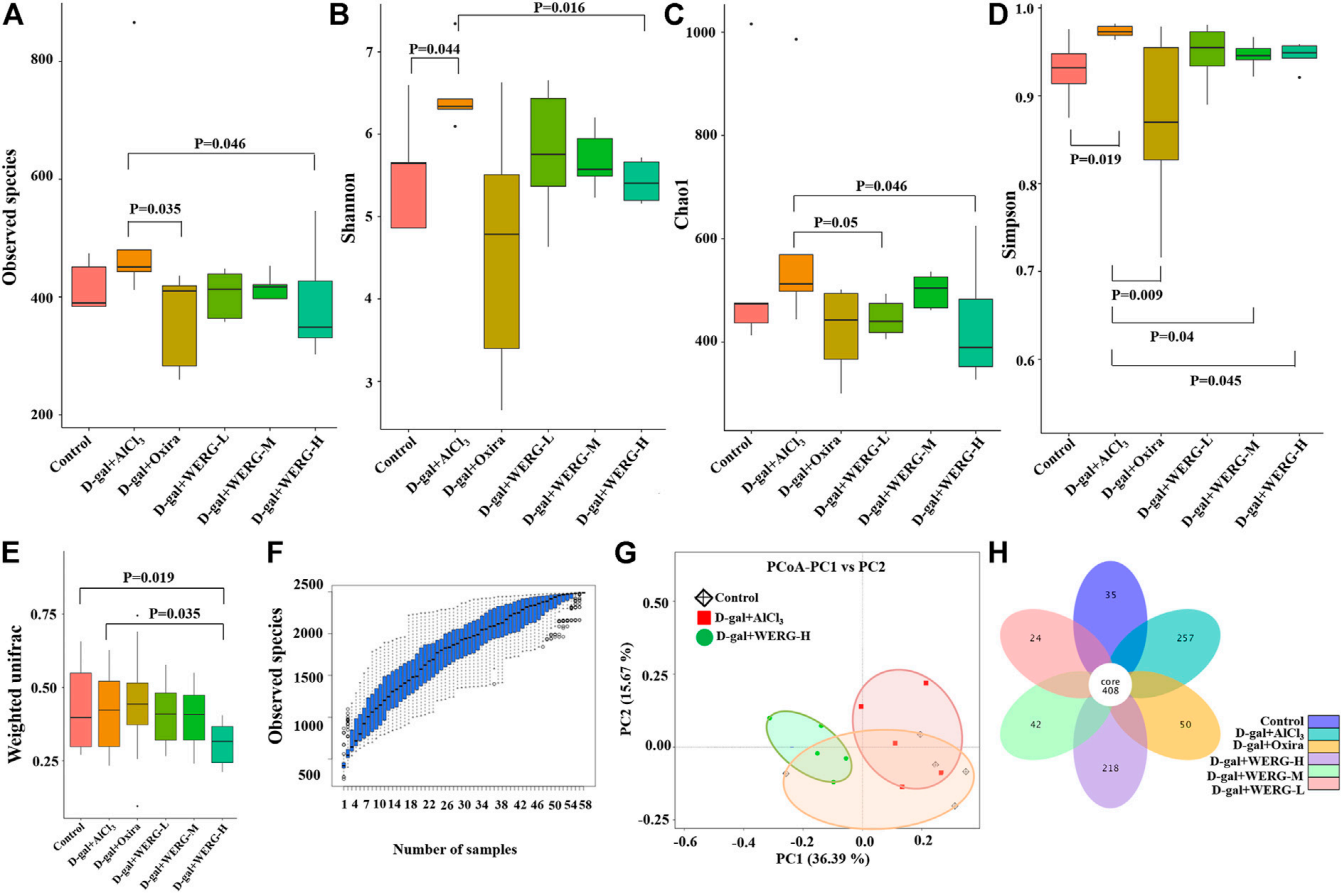
Diversity and richness analysis of WERG-H on gut microbiota in D-gal/AlCl_3_-induced AD mice. The observed species number **(A)**; Shannon diversity index **(B)**; Chao1 diversity index **(C)**; Simpson diversity index **(D)**; weighted UniFrac analysis **(E)**; the species accumulation curve **(F)**; PCoA based on weighted UniFrac distances **(G)**; flower diagrams **(H)**. *n* = 5 mice per group.

Given gut microbiota configurations relate to AD progression; the effect of WERG on the alterations of the intestinal bacterial structure was addressed in WERG-treated mice. Alpha diversity was used to analyze microbial community diversity, which can reflect the richness and diversity of microbial communities within a fecal sample. To assess the effect of WERG on the gut microbiota of D-gal/AlCl_3_-induced AD-like model mice, gut contents were analyzed by 16 S rRNA_V3-4_ gene sequencing. After WERG treatment for a consecutive month, the observed species number of WERG-H (*p* = 0.046) and D-gal + Oxira (*p* = 0.035) groups were reduced significantly compared with the D-gal + AlCl_3_ group (Figure 4A). The Shannon index of control (*p* = 0.044) and D-gal + WERG-H (*p* = 0.016) groups were lower than those of the D-gal/AlCl_3_ group (Figure 4B). The Chao1 index of the WERG-H group also was significantly lower than that of the D-gal + AlCl_3_ group (*p* = 0.046) (Figure 4C). The Simpson index of the control group (*p* = 0.019), D-gal + Oxira (*p* = 0.009), D-gal + WERG-M (*p* = 0.04), and D-gal + WERG-H (*p* = 0.045) groups were significantly decreased compared with that of the D-gal + AlCl_3_ group (Figure 4D). The species accumulation curve (Figure 4F) showed that as the number of samples increases, there will be a greater possibility of discovering a large number of new species; it seems that WERG-H treatment tended to correct the gut microbial disorder tendency. These results indicated that WERG-H treatment could reduce the alpha diversity and abundance of microbes in the D-gal/AlCl3-induced AD mouse model and improve the disturbance of gut microbiota.

Next, the samples were assessed for beta diversity by using PCoA to investigate differences in microbiota composition in the control, D-gal + AlCl_3_, and D-gal + WERG-H groups (Figure 4G), which showed that gut microbial community among the three groups formed distinct clusters. The D-gal + AlCl_3_ and D-gal + WERG-H groups were well separated with 36.39% and 15.67% variation by the principal components PC1 and PC2, respectively. Weighted UniFrac analysis revealed that D-gal + AlCl_3_ treatment drove a marked difference in gut microbiota composition, whereas WERG-H treatment (*p* = 0.035) significantly reduced the alterations (Figure 4E). As shown in Figure 4H, the number of OTUs shared by the six groups is 408, and the number of unique OTUs in the D-gal + WERG-H group was 218. As expected, the petal plot showed that the WERG-H treatment group had more specific OTUs than the other treatment groups.

### WERG Restores Gut Microbiome Imbalances in the AD Mouse Model

**FIGURE 5:**
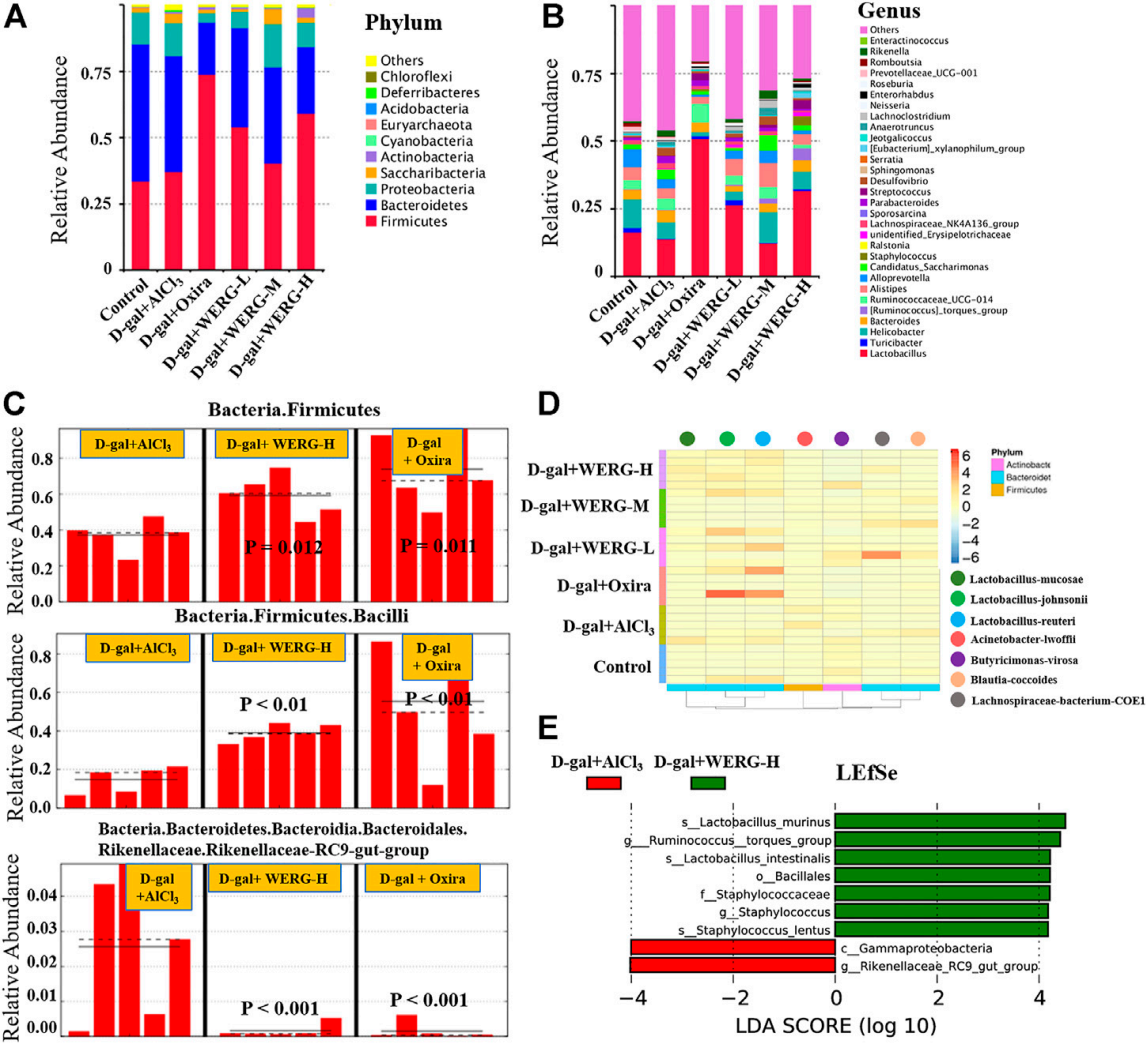
WERG-H alleviated gut microbiota dysbiosis in AlCl_3_/D-gal-induced AD mice. The relative abundance at the phyla level **(A)**; the relative abundance at the genus level **(B)**; biomarker raw images in the sample **(C)**; heatmap analysis of microbial composition **(D)**; LEfSe analysis **(E)**. *n* = 5 mice per group.

The relative abundances at phylum and genus levels were analyzed. The Phylum level analysis revealed that the relative abundance of *Bacteroidetes* was significantly lower in the D-gal + AlCl_3_ group than that in the control group, while the relative abundances of *Saccharibacteria*, *Actinobacteria*, *Cyanobacteria*, *Acidobacteria*, and *Deferribacteres* were significantly increased (Figure 5A). In addition, the relative abundances of *Bacteroidetes*, *Proteobacteria*, and *Saccharibacteria* in the D-gal + Oxira and D-gal + WERG-H groups were significantly decreased compared to those in the D-gal + AlCl_3_ group, while the relative abundances of *Firmicutes* (*p* = 0.012) and *Bacilli* (*p* = 0.011) were significantly increased. The Rikenellaceae-RC9 gut group (*p* < 0.001) was enriched in the D-gal + AlCl_3_ group compared with the treatment group (Figure 5C). The relative abundances at the genus levels were further analyzed (Figure 5B), which showed that the relative abundances of *Lactobacillus*, *Turicibacter*, *Helicobacter*, and *Alloprevotella* were significantly decreased in the D-gal + AlCl_3_ group compared to the control group, while the relative abundances of *Bacteroides*, Ruminococcaceae_UCG-014, *Candidatus_Saccharimonas*, *Staphylococcus*, unidentified_Erysipelotrichaceae, Lachnospiraceae_NK4A136_group, *Sporosarcina*, *Parabacteroides*, *Streptococcus*, *Desulfovibrio*, *Sphingomonas*, *Serratia*, *Jeotgalicoccus*, *Anaerotruncus*, *Roseburia*, *Rikenella*, and *Enteractinococcus* were increased. However, the relative abundances of *Bacteroides, Candidatus_Saccharimonas*, Lachnospiraceae_NK4A136_group, *Sporosarcina*, *Parabacteroides*, and *Desulfovibrio* have decreased in the D-gal + Oxira and D-gal + WERG-H groups compared to the D-gal + AlCl_3_ group, while the relative abundances of *Lactobacillus*, *Turicibacter*, and *Staphylococcus* were markedly increased. As shown in Figure 5D, the effect of WERG treatment on the relative abundance of gut microbial taxa in an AD mouse model was analyzed according to MetaStat. The relative abundances of *Lactobacillus-mucosae*, *Lactobacillus-johnsonii*, and *Lactobacillus-reuteri* in the D-gal + WERG-H group were increased compared with the other treatment group. Then, a linear discriminatory analysis (LDA) effect size (LEfSe) analysis was performed to further determine whether specific individual bacterial taxa were differentially enriched in the D-gal + WERG-H group. As shown in Figure 5E, this analysis identified nine genera, which were differentially abundant between the D-gal + WERG-H and D-gal + AlCl_3_ groups. The results showed that *s-Lactobacillus-murinus*, *g-Ruminococcus-torques-group*, *s-Lactobacillus-intestinalis*, *o-Bacillales*, *f-*Staphylococcaceae, *g-Staphylococcus*, and *s-Staphylococcus-lentus* were enriched in the WERG-H treatment group.

### WERG-H Modulated Specific Phylotypes of Gut Microbiome and Increased the Probiotic Species in the AD Mouse Model

**FIGURE 6:**
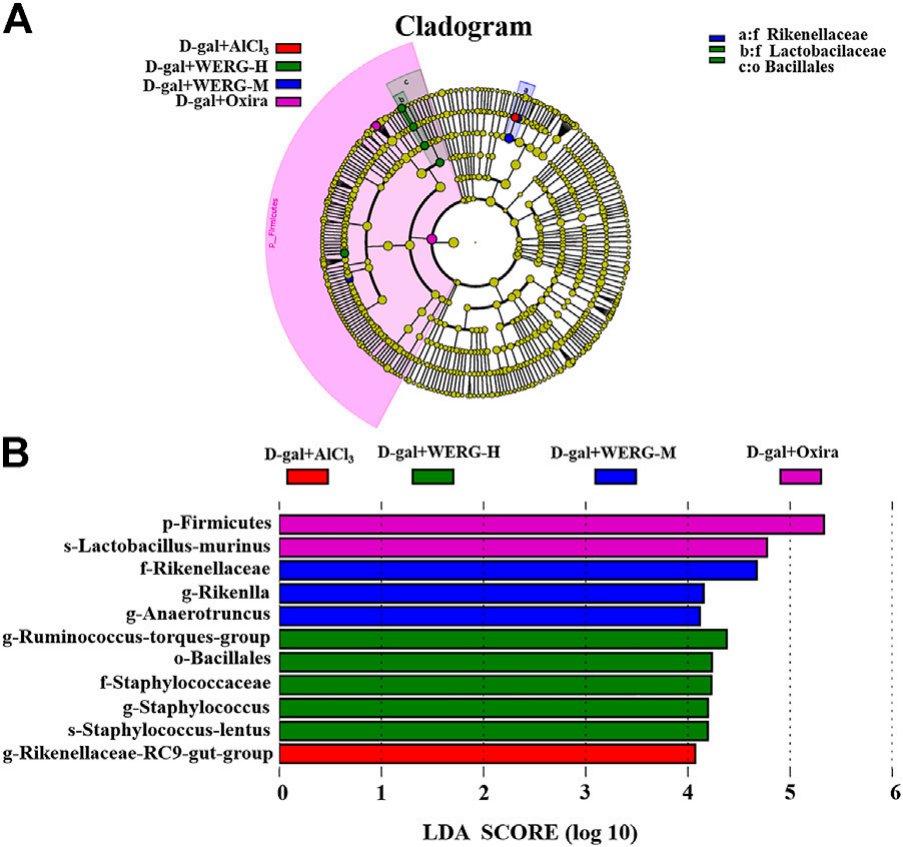
Gut microbiota differences. Cladograms reveal microbial phylogenetic branches associated with treatment groups status in the AD mouse model **(A)**; linear discriminant analysis (LDA) **(B)**. Statistical significance reflects both *p* < 0.05 for Student’s t test and LDA score threshold > 4 was listed, *n* = 5 mice per group.

**FIGURE 7:**
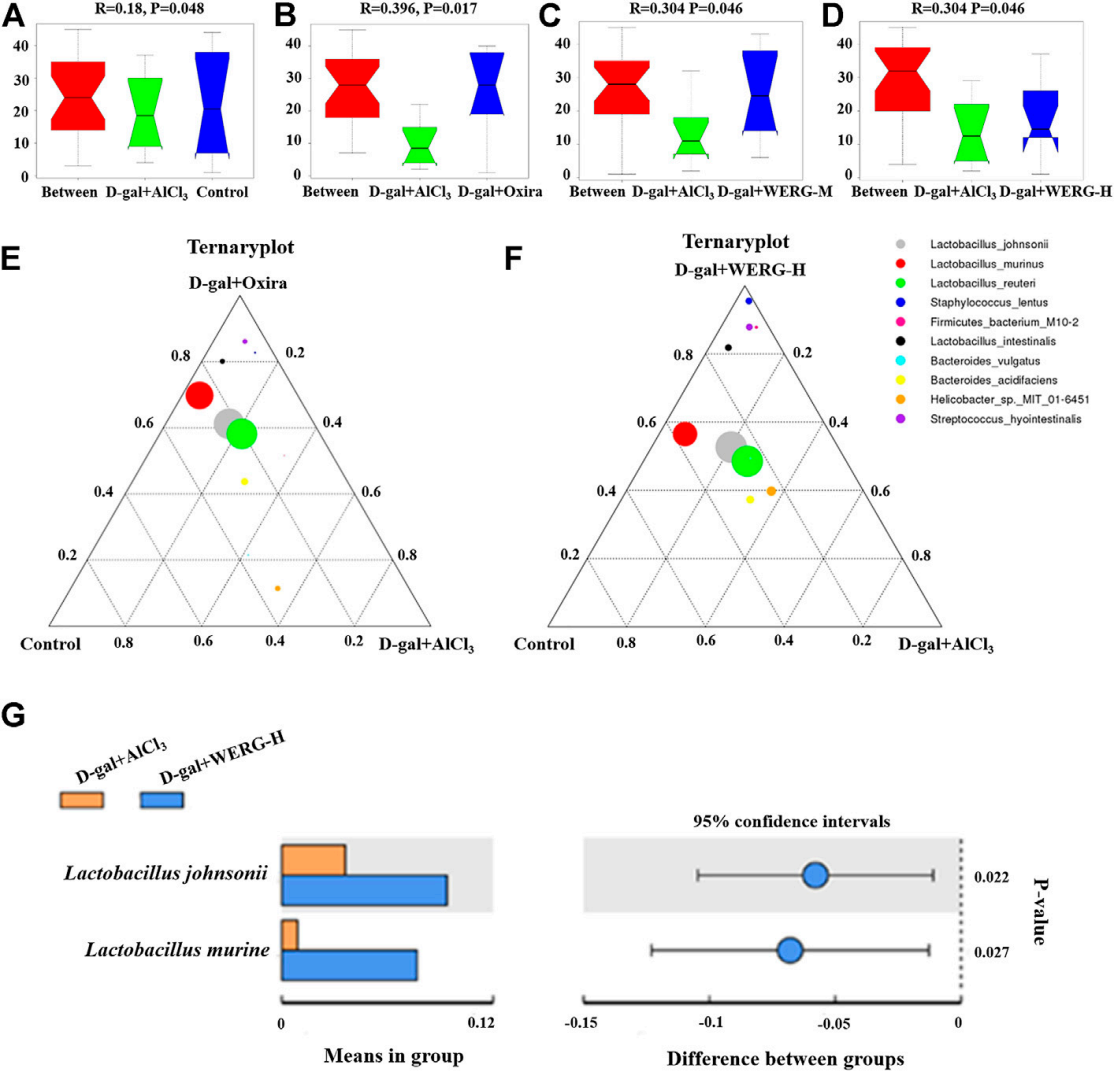
WERG-H treatment increased the probiotic species. Anosim analysis results **(A–D)**; ternary plot **(E,F)**; *t*-test analysis **(G)**. Significant statistical difference by Student’s t-test (*p* < 0.05). *n* = 5 mice per group.

LEfSe analysis was further performed to identify statistically significant biomarkers of gut microbiota in different groups. The Linear discriminatory analysis (LDA) score distribution histogram (based on LDA score > 4) and Cladograms analysis were conducted, and a series of biomarkers were identified as shown by the cladogram (Figures 6A,B). A total of 11 OUTs were notably different among all groups. In the D-gal + Oxira group, there were two OUTs and the *p-Firmicutes* and *s-Lactobacillus-murinus* were the obvious difference. The three OUTs of f-Rikenellaceae, *g-Rikenlla*, and *g-Anaerotruncu* showed remarkable differences in the D-gal + WERG-M group. The most prominent different features in the D-gal + WERG-H group were five OUTs, namely, g-*Ruminococcus-torques-group*, *o-Bacillales*, *f-Staphylococcaceae*, *g-Staphylococcus*, and *s-Staphylococcus-lentus*. There was a g-Rikenellaceae-RC9 gut group that exhibited a conspicuous difference in the D-gal + AlCl_3_ group. It can be seen from Figures 7A–D that there are significant differences in colony distribution between the control group and the D-gal + AlCl_3_ group (*p* = 0.048), the D-gal + Oxira group and the D-gal + AlCl_3_ group (*p* = 0.017), the D-gal + WERG-M group and the D-gal + AlCl_3_ group (*p* = 0.046), and the D-gal + WERG-H group and the D-gal + AlCl_3_ group (*p* = 0.046). Ternary plot analysis was used to display common flora or OTUs in three groups. The distribution of species in the D-gal + Oxira and D-gal + WERG-H groups was further analyzed using ternary plots. The results showed that the main enriched species include *Lactobacillus-johnsonii*, *Lactobacillus-murinus*, *Lactobacillus-reuteri*, *Staphylococcus-lentus*, *Firmicutes-bacterium-M10-2*, *Lactobacillus-intestinalis*, *Bacteroides-vulgatus*, *Bacteroides-acidifaciens*, *Helicobacter-sp-MIT-01–6451*, and *Streptococcus-hyointestinalis*. In general, the three probiotics of *Lactobacillus-johnsonii*, *Lactobacillus-murinus*, and *Lactobacillus-reuteri* were significantly enriched in the D-gal + Oxira and the D-gal + WERG-H groups, which were located in the upper part of the ternary graph (Figures 7E,F). Multiple comparisons were further corrected to show significant differences between D-gal + AlCl_3_ and D-gal + WERG-H groups at the species levels (Figure 7G). When compared with the D-gal + AlCl_3_ group, the *Lactobacillus-johnsonii* (*p* = 0.022) and *Lactobacillus-murinus* (*p* = 0.027) were significantly enriched in the D-gal + WERG-H group.

## Discussion

Currently, amyloid plaques (Aβ) and neurofibrillary tangles (p-Tau) are two typical pathological features in AD pathogenesis (West and Bhugra, 2015). However, the pathogenesis of AD remains unclear. In this study, our main findings are the associations between gut microbiota composition and p-Tau^Thr231^ status. To our knowledge, we are the first to report an association between this microbe and AD biomarkers. Tau hyperphosphorylation causes most tau lesions including AD (Mazanetz and Fischer, 2007). Hyperphosphorylated tau was accumulated in the intracellular region and caused neurofibrillary tangles, dysregulated neuronal excitability (Hatch et al., 2017), impaired synaptic plasticity, and neurotransmittance, thus inducing learning and memory impairments. Due to the limited efficiency of new drugs for clearing β-amyloid in AD, tau protein has received more attention as a promising therapeutic target (Panza et al., 2019).

Gut microbiota composition was associated with amyloid and p-tau status. For instance, the abundance of SCFA-producing microorganisms is inversely proportional to the positive rate of amyloid and p-tau status (Verhaar et al., 2021). Animal studies have reported significantly reduced SCFA-producing microbes in AD mice when compared to wild-type mice (Zhang et al., 2017; Sun et al., 2019). Transplantation of fecal microbiota from wild-type mice to APP/PS1 and ADLPAPT mice resulted in a reduction in amyloid, suggesting a causal link between gut microbes and AD (Sun et al., 2019; Kim et al., 2020). In addition, an SCFA, sodium butyrate intervention can reduce AD pathology (Fernando et al., 2020). In this study, we developed a novel tau-based therapeutic strategy, which may provide early treatment of AD and related tau lesions before abnormal tau accumulation.

Inflammation and oxidative damage, two potential triggers for AD symptoms, can cause brain damage and induce impairments in synaptic function and memory (Zheng et al., 2019). D-gal/AlCl_3_ can cause oxidative stress damage, and further develop many other dysfunctions of the central nervous system by generating ROS and inducing neurodegeneration (Rehman et al., 2017; Wang et al., 2019). Previous studies have shown that D-gal/AlCl_3_ can cause decreased memory and learning abilities, Aβ deposition, and enhanced p-tau expression, and provide an effective non-transgenic AD-like injury model (Zhang et al., 2016a; An et al., 2017; Chiroma et al., 2018). This study mainly investigated the neuroprotective effect of WERG in D-gal/AlCl_3_-induced AD mice. Substantially, WERG-H significantly alleviated D-gal/AlCl_3_-stimulated cognitive impairment, p-Tau^Thr231^ protein formation, and pathological changes. Consistent with the present study, gastrodin significantly inhibited lead-induced p-Tau accumulation in the mouse brain (Liu et al., 2020). Similarly, another study confirmed that gastrodin suppressed the deposition of p-Tau in the brain of the unilateral intracerebroventricular injection of the Aβ_1-42_ mouse model (Luo et al., 2022). Moreover, WERG treatment reduced corticosterone, adrenocorticotrophic hormone, hypothalamic corticotropin-releasing factor, and glucocorticoid receptor levels, and decreased plasma interleukin-1β, interleukin-6, and tumor necrosis factor-α concentrations (Wang et al., 2020a). Rhizoma Gastrodiae powder can significantly improve the learning and cognitive ability of mice in the radiation water maze, and the learning and memory impairment in aluminum chloride-induced rats (Shuchang et al., 2008; Mishra et al., 2011). Rhizoma Gastrodiae water extract (WERG) can improve the learning and memory impairment caused by forced swimming in rats and shorten the dark avoidance latency and platform-seeking time of rats in the MWMT (Chen et al., 2011). Accumulating research suggested that WERG can improve memory and learning cognitive dysfunction (Hu et al., 2014; Park et al., 2015; Liu et al., 2018).

**FIGURE 8:**
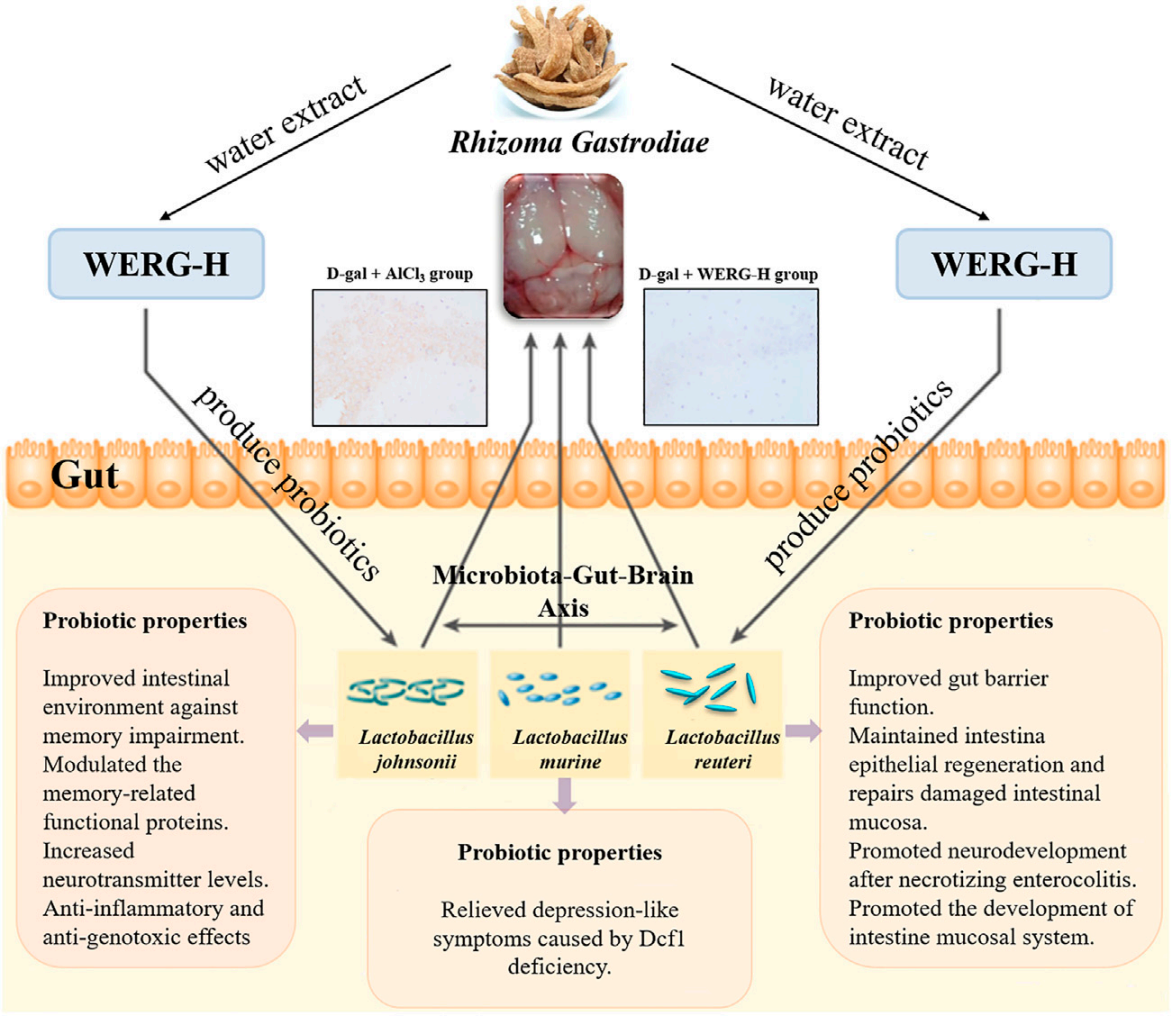
Beneficial effects of WERG-H against AD model mouse may be due to inhibition of p-Tau^Thr231^ protein expression, amelioration of p-Tau^Thr231^-induced toxicity, and alleviation of gut microbiota dysbiosis by enriching probiotics. Finally, through the microbiota–gut–brain axis to improve D-gal/AlCl_3_-induced cognitive impairment.

Gut microbiota exerted an important influence on the progression of AD. Gut barrier permeability may be altered by exogenous or endogenous factors as a consequence of the inflammatory process in AD (Wang et al., 2020b). In short, a decreased number of beneficial bacteria and an increased number of pathogenic bacteria led to a disturbance in the composition of the gut microbiota in AD mice. LEfSe can be used to find biomarkers of differences between groups in high-dimensional data. In this study, the D-gal/AlCl_3_ treatment group had differential biomarkers in the g-Rikenellaceae-RC9 gut group (*p* < 0.001) and *c-Gammaproteobacteria* (*p* < 0.05) at the genus level. A previous study suggested that several specific differential biomarkers were found to be significantly associated with improvements in host parameters, and linolenic acid ameliorated HFD-induced multi-tissue metabolic disorders and gut microbiota disorders. Among them, the Rikenellaceae-RC9 gut group was positively correlated with HFD-induced harmful indicators and negatively correlated with a beneficial indicator (Gao et al., 2020). At the genus level, the Rikenellaceae-RC9 gut group was more abundant in fecal samples from PD patients (Yan et al., 2021). In addition, another study showed that *c-Gammaproteobacteria* gradually increased from healthy control patients to amnestic mild cognitive impairment patients and then AD patients (Liu et al., 2019). These results provide a preliminary basis for the mining of biomarkers in AD. It has been reported that the abundance of pathogenic bacteria is increased, while the abundance of beneficial bacteria is decreased in Aβ_42_-induced AD mice (Xu et al., 2020). In the present study, our results showed that the WERG-H treated group had differential biomarkers in *L. johnsonii* (*p* = 0.022) and *Lactobacillus murine* (*p* = 0.027), and enriched in *Lactobacillus-reuteri*. The above results showed a stimulatory effect of water extracts from Gastrodiae Rhizoma on probiotic growth at optimal dosage. The D-gal + Oxira group is also mainly enriched in *Lactobacillus-johnsonii*, *Lactobacillus-murinus*, and *Lactobacillus-reuteri*. Studies have reported that the traditional Chinese medicine prescription “Huanglian Jiedu Decoction” can reverse the cognitive impairment of Tg mice and increase *Bacteroides* S24-7 and *Lactobacillus* in Tg mice (Gu et al., 2021). As shown in Table 2, many studies have reported the beneficial effects of probiotics on neurological disorders and gut microbiota. A previous study showed that *L. johnsonii* BS15 intake can improve intestinal inflammation, neuroinflammation, and fluorine-induced and restraint stress-induced memory dysfunction by improving inflammation and permeability (Xin et al., 2020; Wang et al., 2021). In addition, *L. johnsonii* CJLJ103 was able to alleviate colitis and memory impairment by inhibiting NF-jB activation and intestinal lipopolysaccharide production (Lim et al., 2017). *L. johnsonii* 456 is associated with reduced inflammation and genotoxicity in vertebrate models (Davoren et al., 2019). Furthermore, probiotic *L. johnsonii* L531 can promote SCFA production to control *Salmonella* infection (He et al., 2019). *L. murine* and *L. reuteri* intestinal transplantation improved depression-like symptoms caused by Dcf1 deficiency (Zhou et al., 2022). These results suggest that WERG-H treatment can ameliorate intestinal metabolic disturbances by increasing the abundance of probiotics, thereby exerting anti-AD effects by remodeling the gut microbiota and reducing p-tau levels (Figure 8). Regrettably, this study has not yet explored the active ingredients that play a major role in WERG, and will focus on the research on the active ingredients and their mechanism of action on AD later.

**TABLE 2:** Effects of probiotics on neurological disorders and gut microbiota.

Probiotics	Subject	Effects	Reference
*Lactobacillus johnsonii* BS15	Fluoride-induced mice	Improved intestinal environment and improved memory impairment	Xin et al. (2020)
*Lactobacillus johnsonii* BS15	Mice	Modulated memory-related proteins and increased neurotransmitter levels	Wang et al. (2021)
*Lactobacillus*, *Helveticus* R0052	Mice	Reduce anxiety and improve memory	Ohland et al. (2013)
*Lactobacillus plantarum* MTCC 1325	D-Galactosea-induced AD-like rat model	Improved acetylcholine levels, prevented Aβ plaque formation, and improved cognitive function	Nimgampalle and Kuna, (2017)
*Lactobacillus*, *Helveticus*, *Lactobacillus Rhamnosus*	Diabetic rat	Improved spatial memory impairment	Davari et al. (2013)
*Lactobacillus casei* strain *Shirota* (LcS)	*In vivo* mouse model of EAE	Reduced neuroinflammation	Kobayashi et al. (2012)
*Lactobacillus*	Aβ-Induced AD rat model	Improved memory, learning abilities, and oxidative stress	Everard et al. (2013)
*Clostridium butyricum*	Mice	Improved neuronal apoptosis and histopathological changes	Liu et al. (2015)
*Bifidobacterium breve strain* A1	Aβ-Induced mice	Blocked Aβ-induced cognitive impairment	Kobayashi et al. (2017)
*Lactobacillus johnsonii* CJLJ103	Mouse	Anti-colitic and memory ameliorating effects	Lim et al. (2017)
*Lactobacillus murine* and *Lactobacillus reuteri*	Mouse	Depression-like symptoms caused by Dcf1 deficiency were relieved	Zhou et al. (2022)
*Lactobacillus johnsonii* 456	Mouse	Anti-inflammatory and anti-genotoxic effects	Davoren et al. (2019)
*Lactobacillus brevis* FP A3709	Sprague–Dawley rats	Antidepressant effects	Ko et al. (2013)
*Lactobacillus helveticus* Bar13	Healthy adults	No increase in *Clostridium* cluster XI	Rampelli et al. (2013)
*Lactobacillus casei*	Healthy adults	Altered the diversity and composition of the gut microbiota	Zhang et al. (2014)
*Lactobacillus paracasei* DG	Healthy adults	Increased in Proteobacteria and *Coprococcus*; decreased in *Blautia*	Ferrario et al. (2014)
*Lactobacillus johnsonii* L531	Pigs	*Salmonella colonization* levels were significantly reduced	He et al. (2019)
*Lactobacillus salivarius* UBLS22	Healthy adults	Increase in *lactobacilli* and decrease in *E. coli*	Rajkumar et al. (2015)
*Lactobacillus casei* NCDC 19	Mice	Increase in *bifidobacteria* population	Rather et al. (2014)
*Lactobacillus reuteri*	Mouse	Improve gut barrier function	Li et al. (2019)

## Conclusion

Gut microbiota composition was associated with p-tau status. Our study showed observed associations between *L. johnsonii*, *L. murine*, and *Lactobacillus-reuteri* levels and AD biomarkers by showing that higher abundances of probiotic microbes were associated with lower odds of positive p-tau status.

## Data Availability

The datasets presented in this study can be found in online repositories. The names of the repository/repositories and accession number(s) can be found below: https://www.ncbi.nlm.nih.gov/, PRJNA541119, PRJNA541132, PRJNA541161, PRJNA541165.
