# Supplementary figures and images for: Rhizoma Gastrodiae Water Extract Modulates the Gut Microbiota and Pathological Changes of P-TauThr231 to Protect Against Cognitive Impairment in Mice
Source: Front Pharmacol. 2022 Jul 15;13:903659. doi: 10.3389/fphar.2022.903659 (PMC9335362; doi:10.3389/fphar.2022.903659)

HE


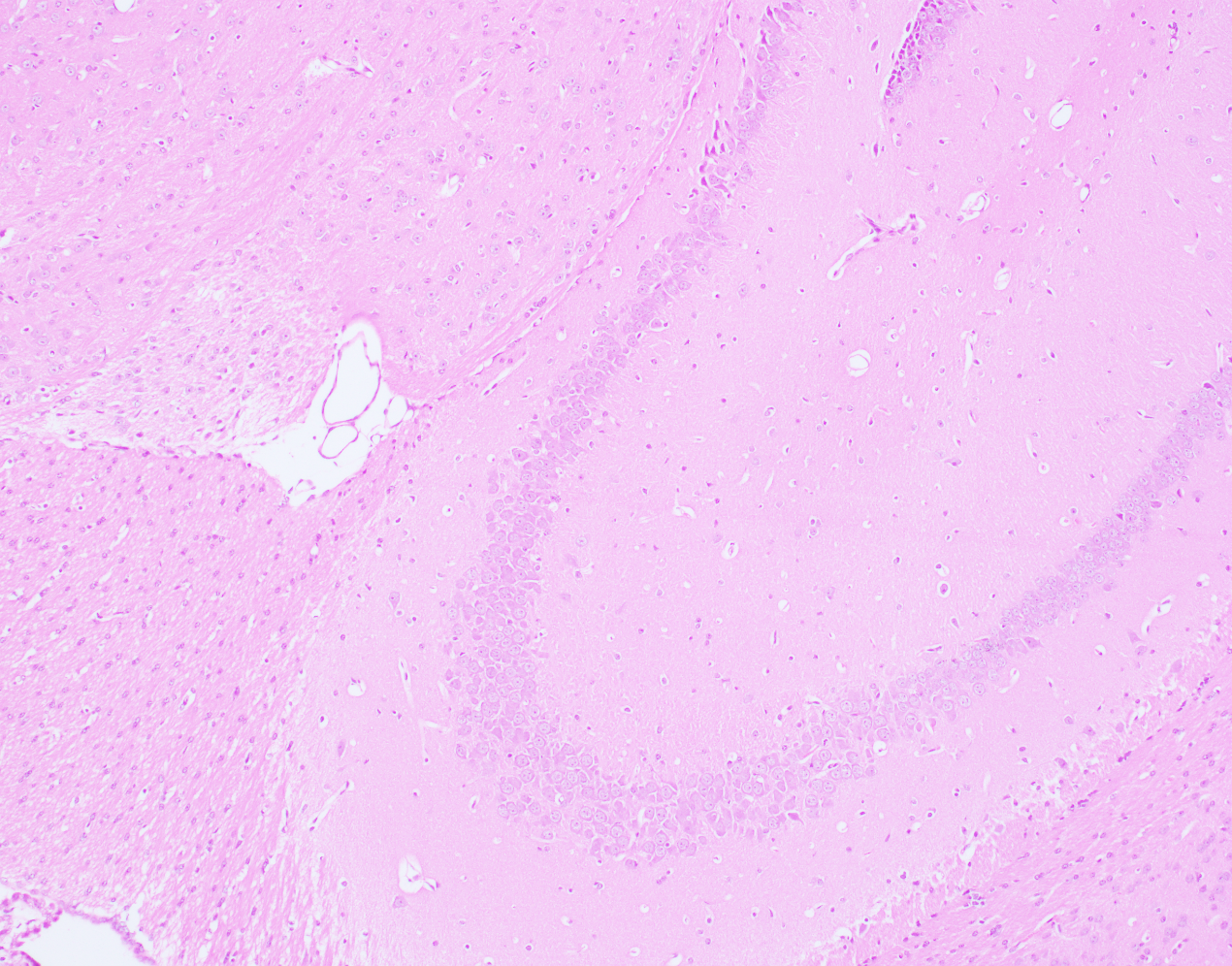

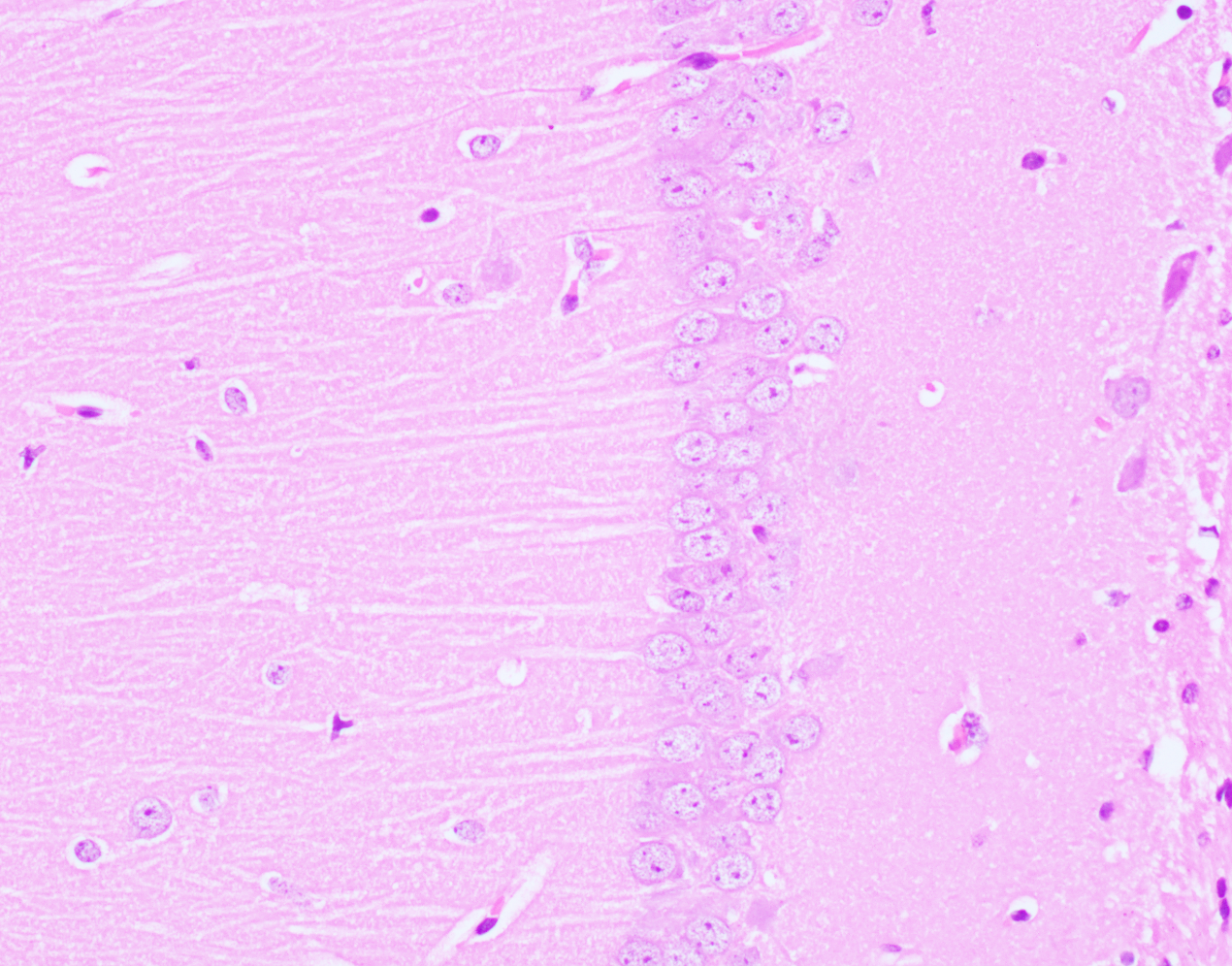

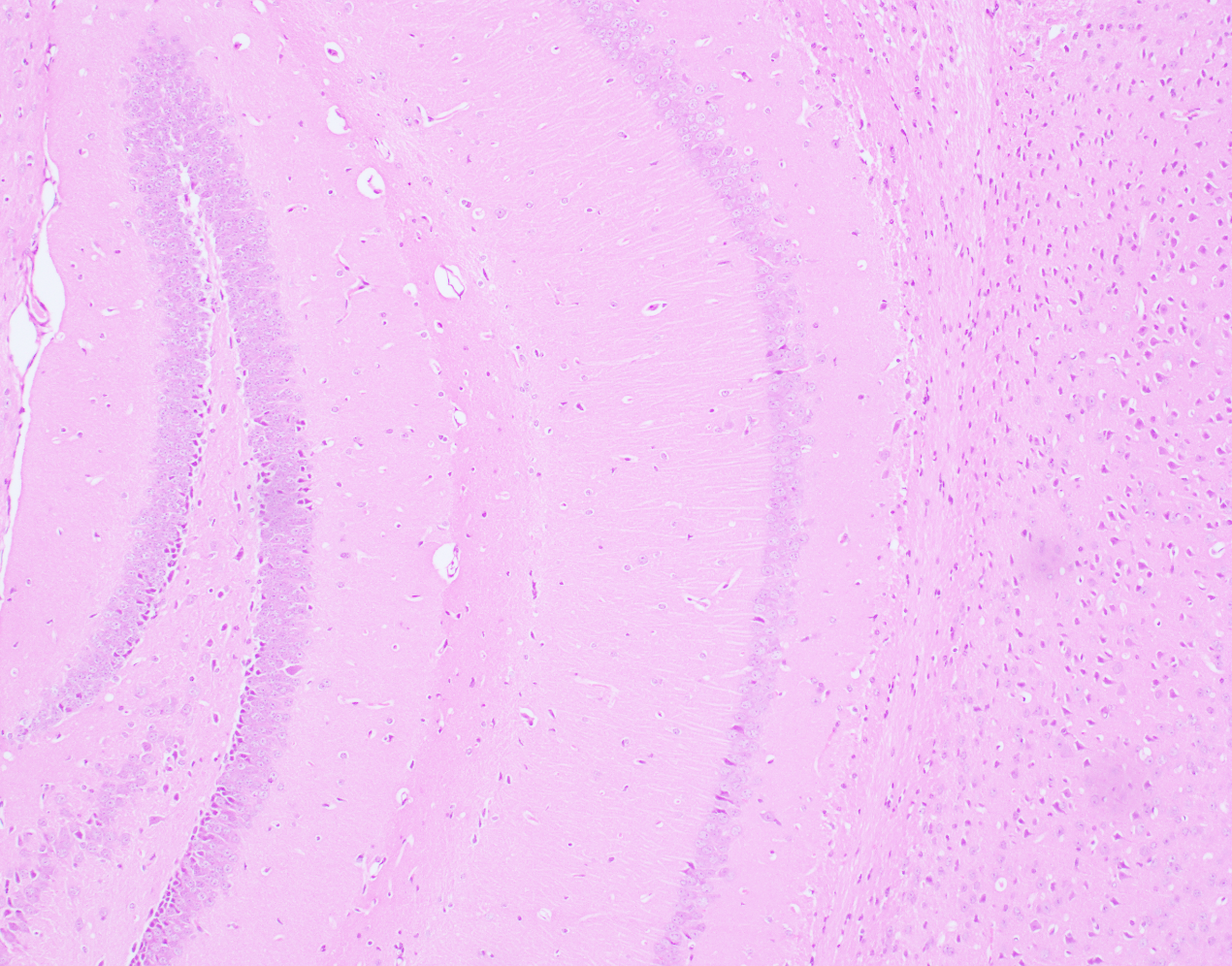

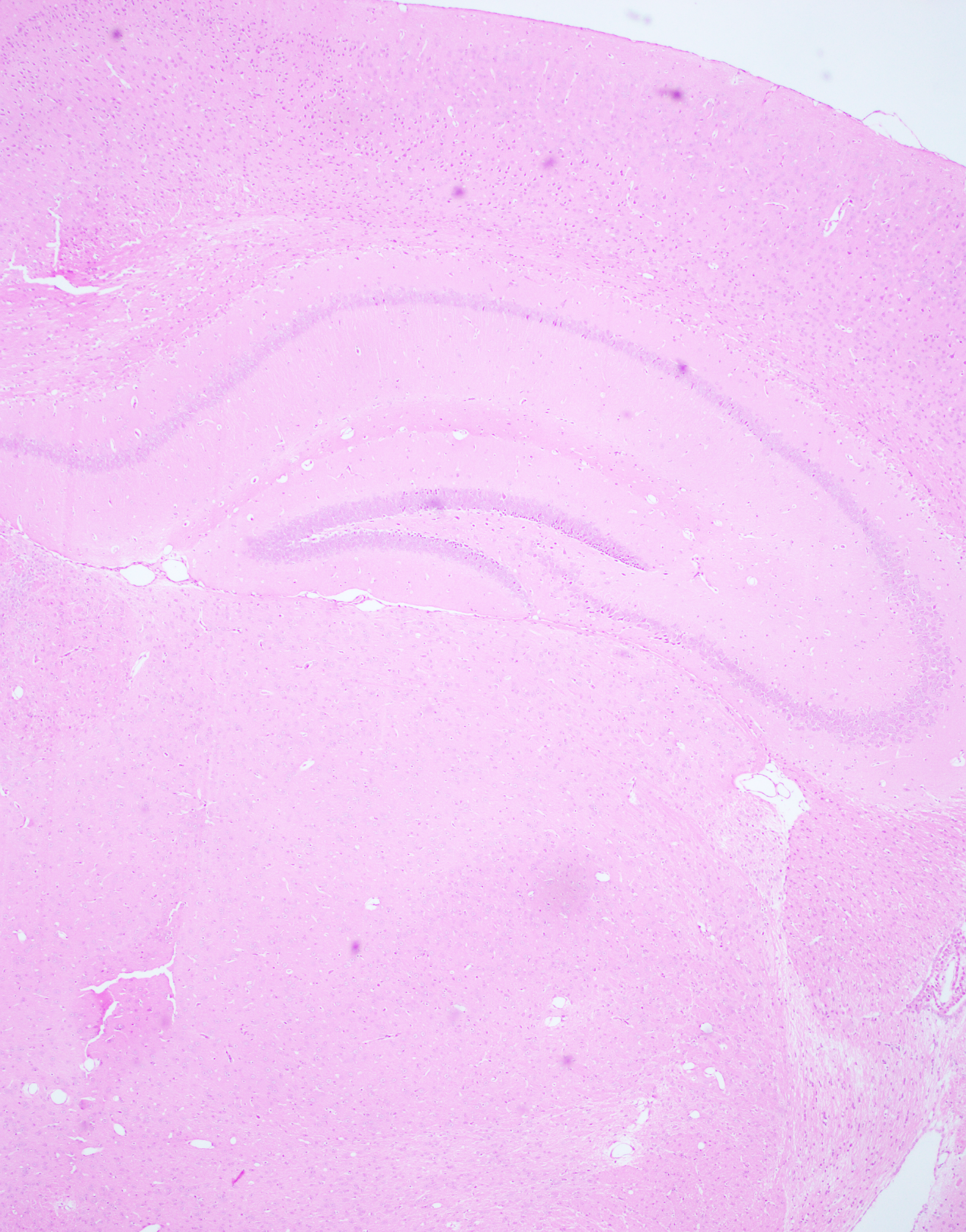

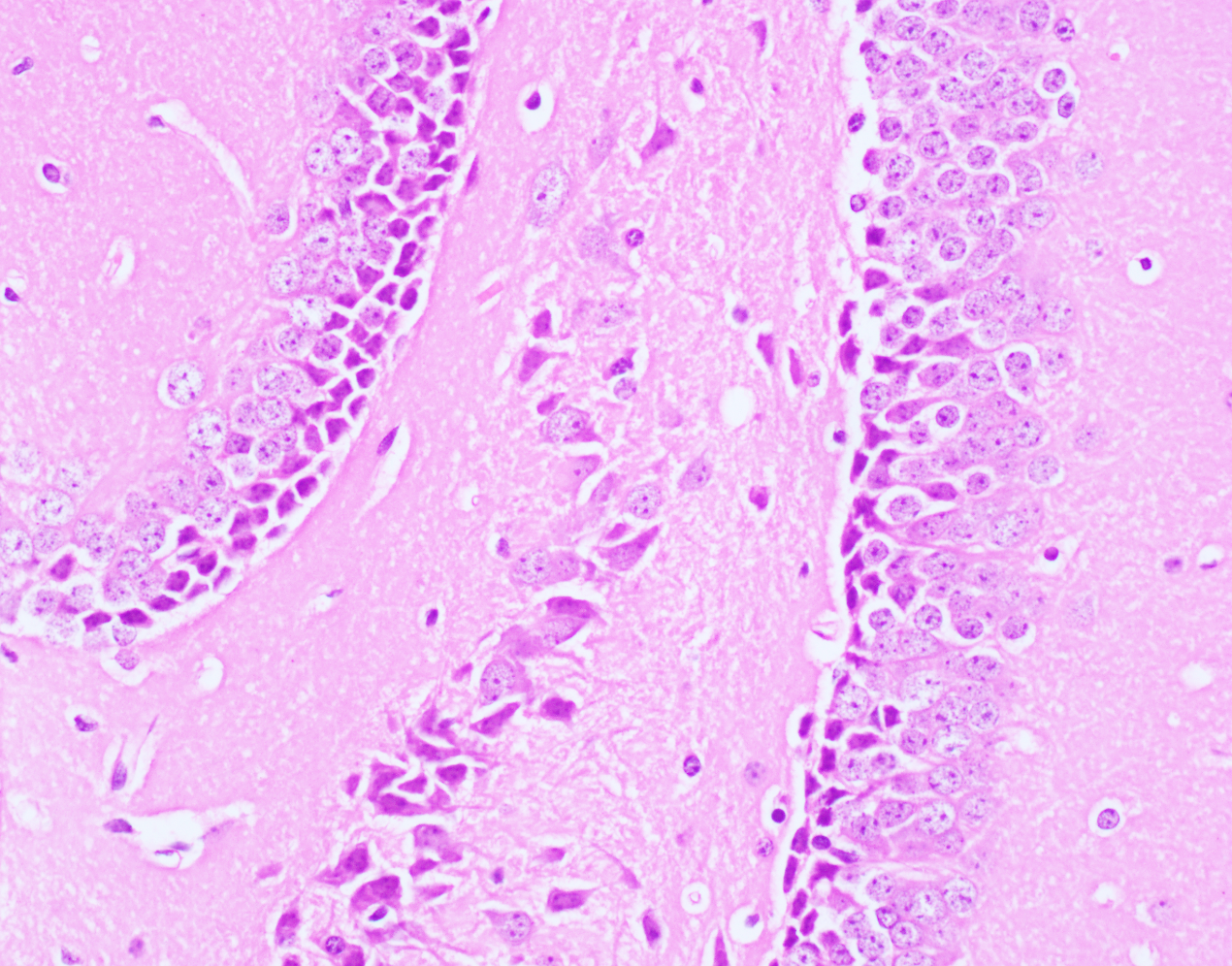

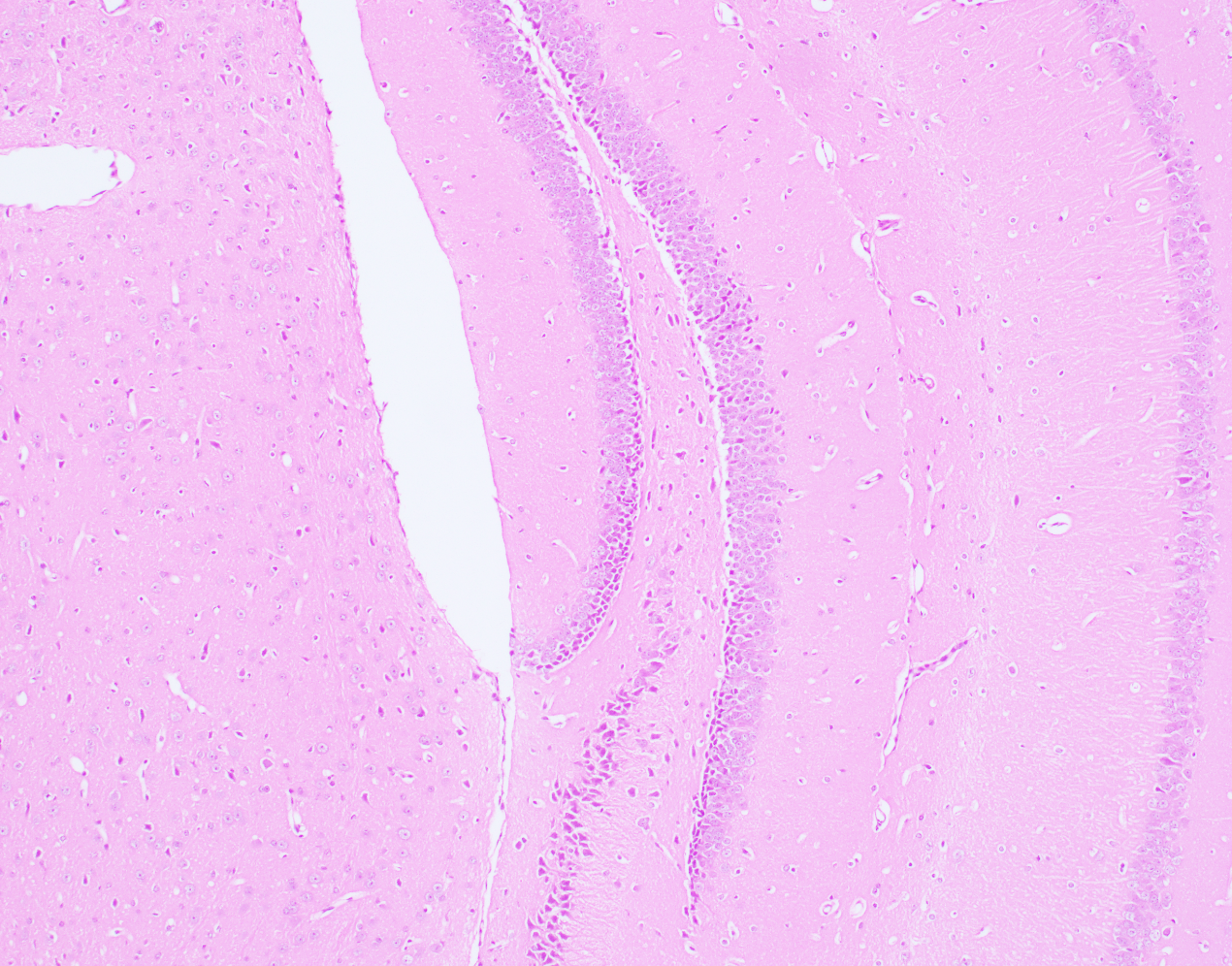

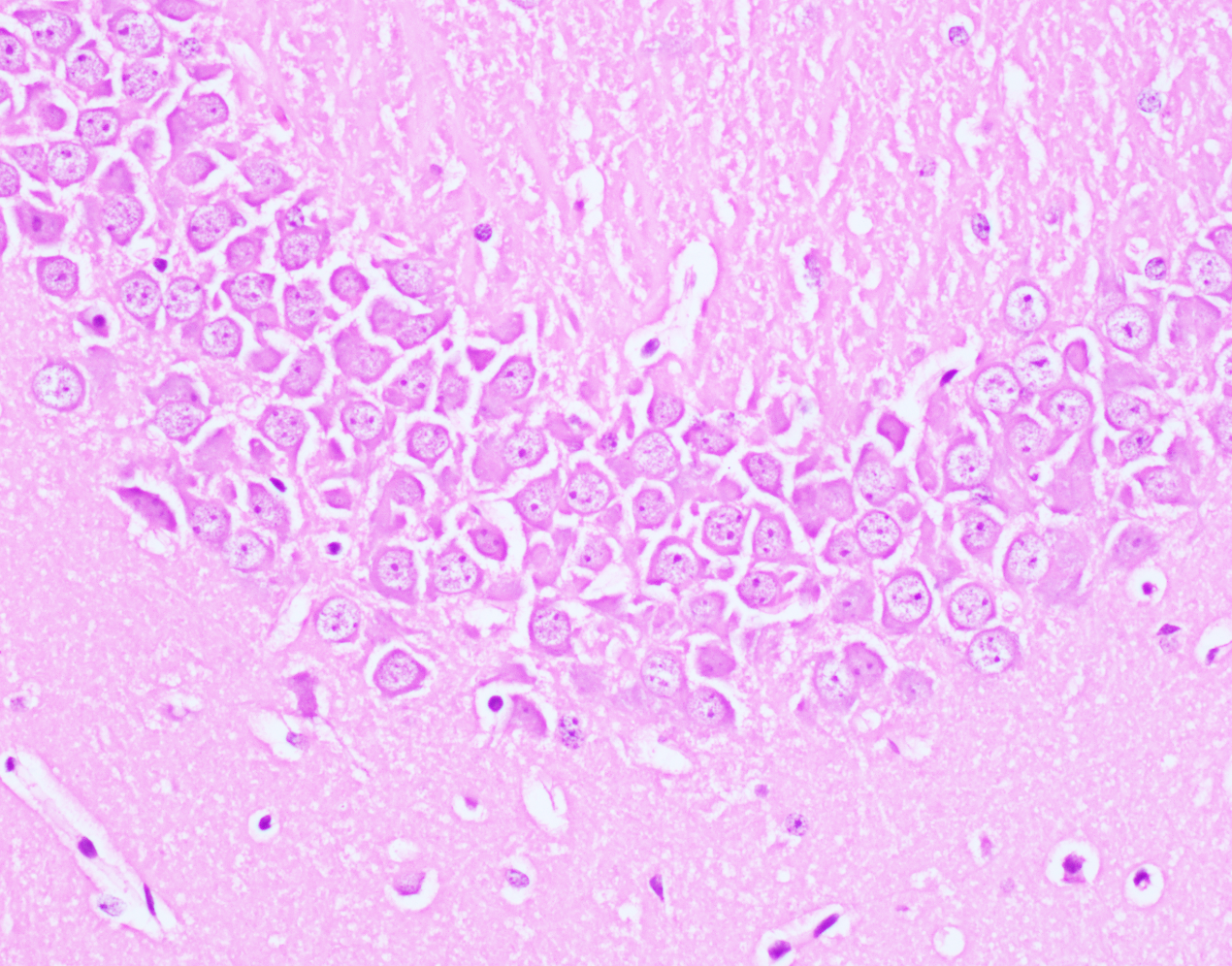

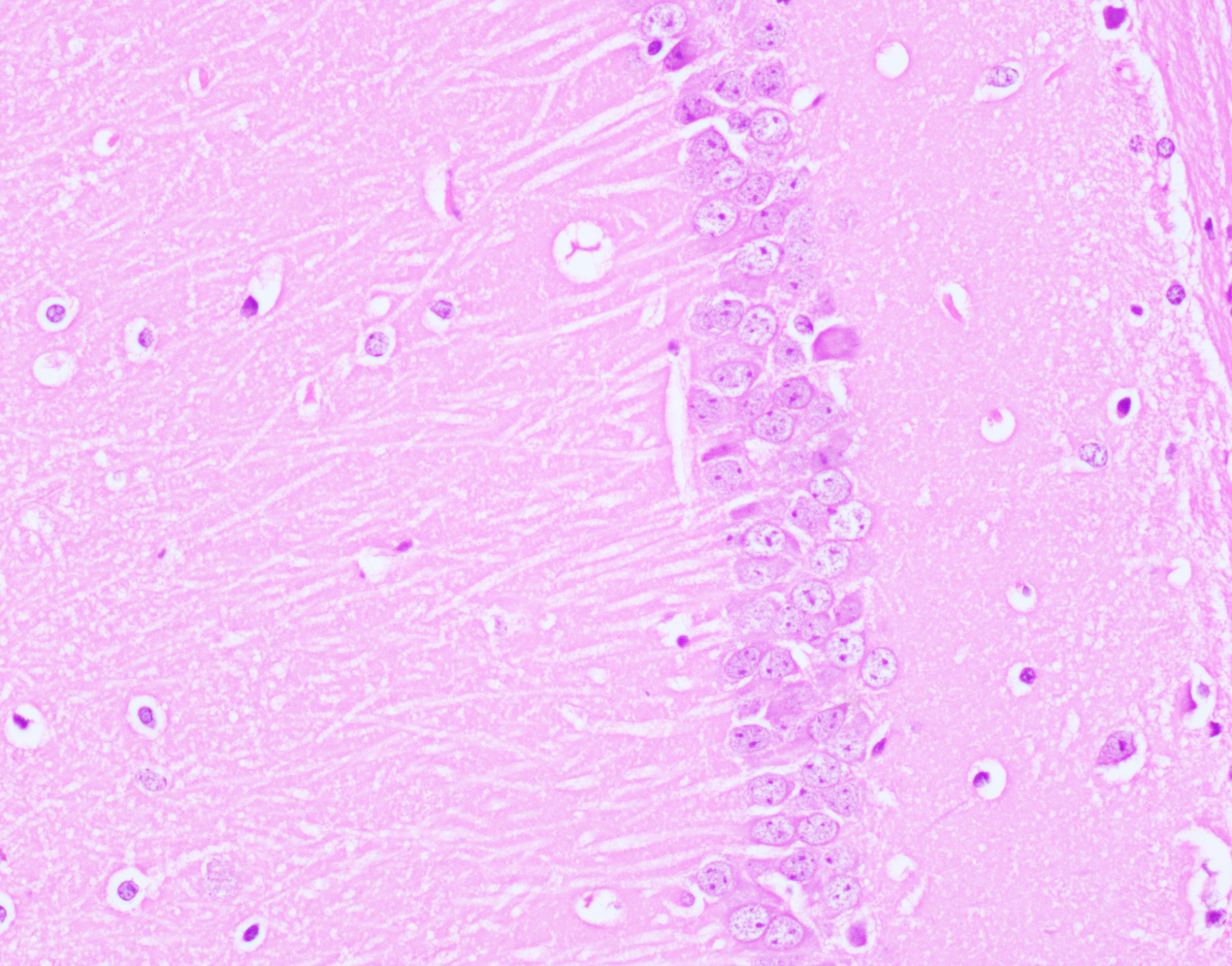

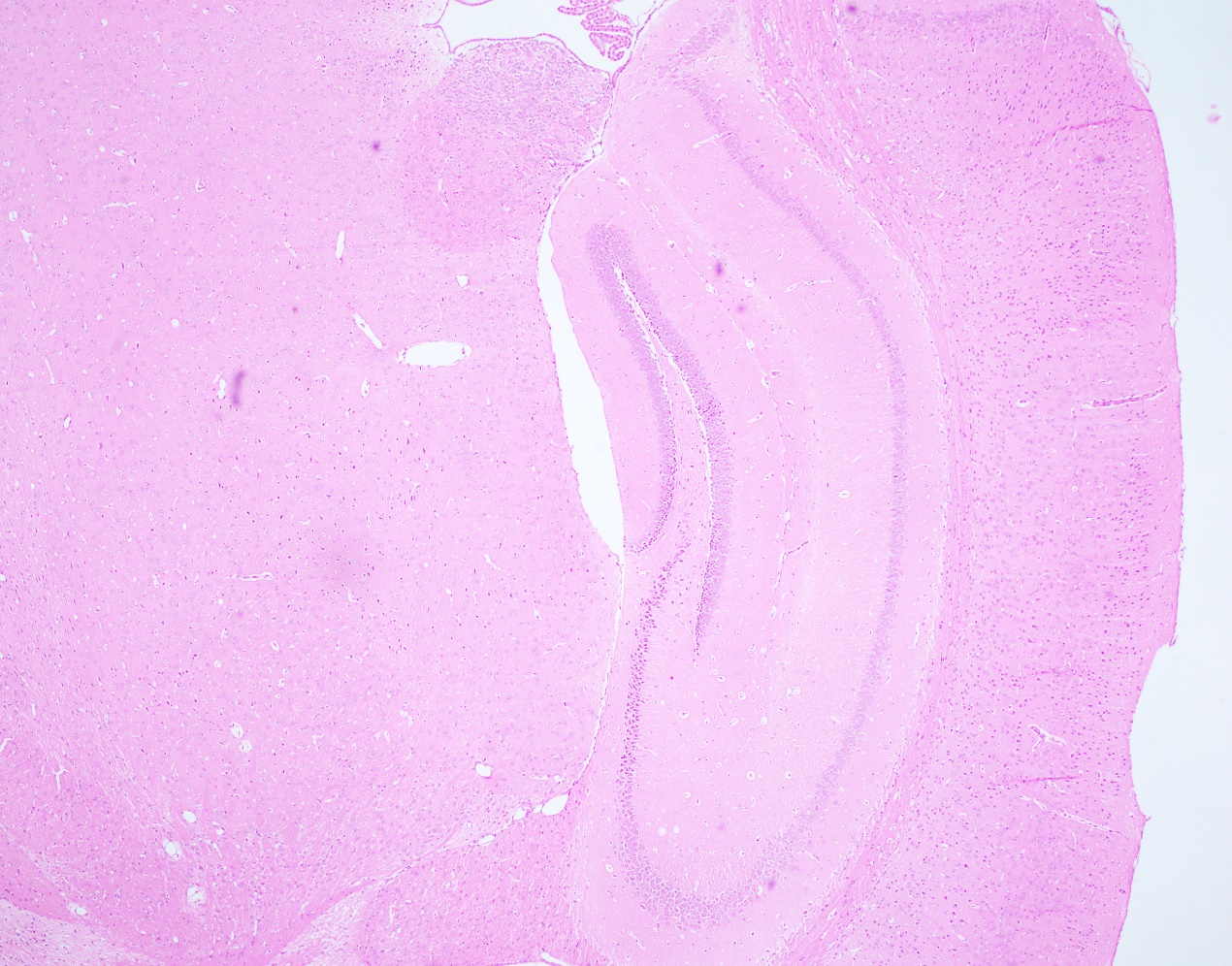

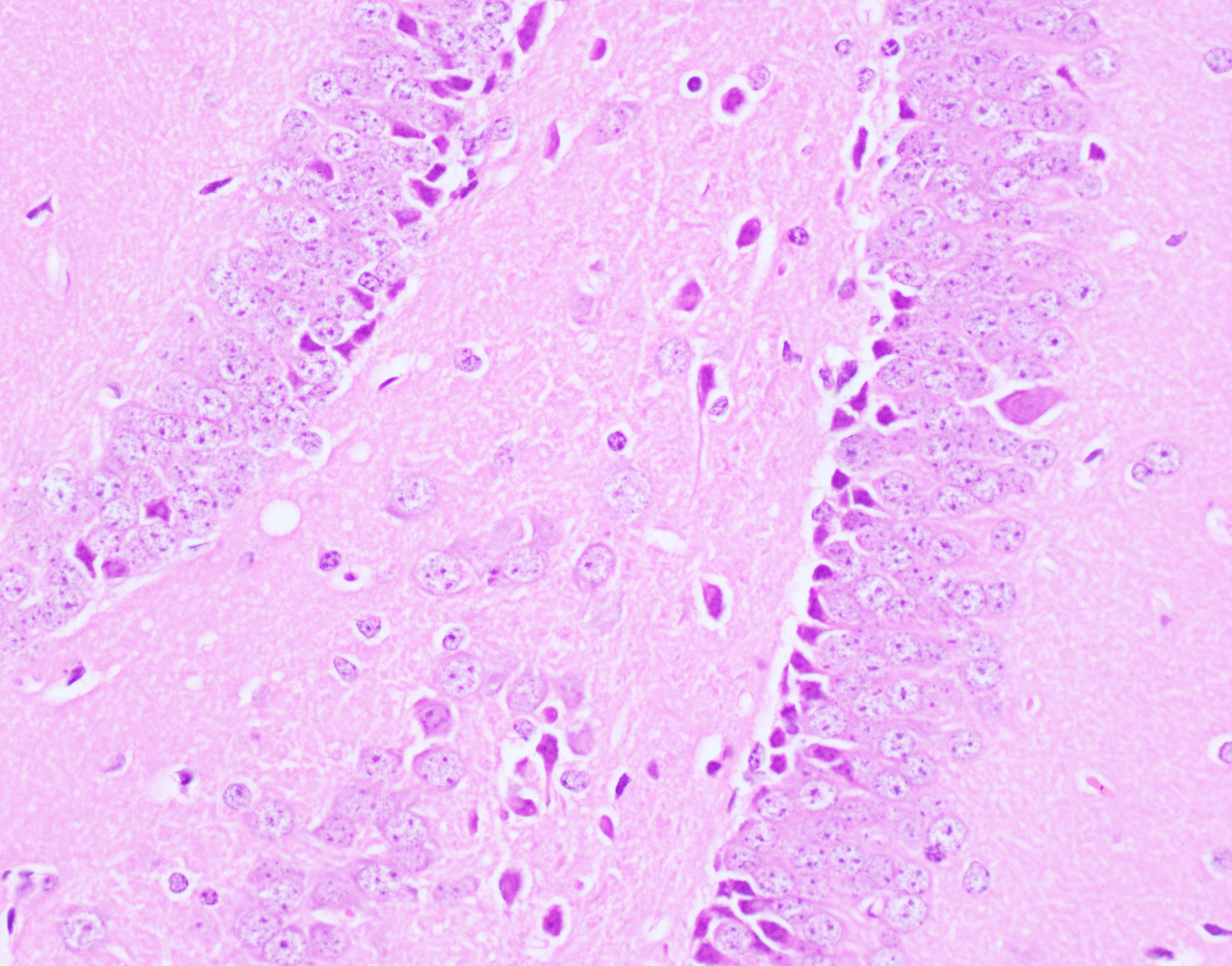

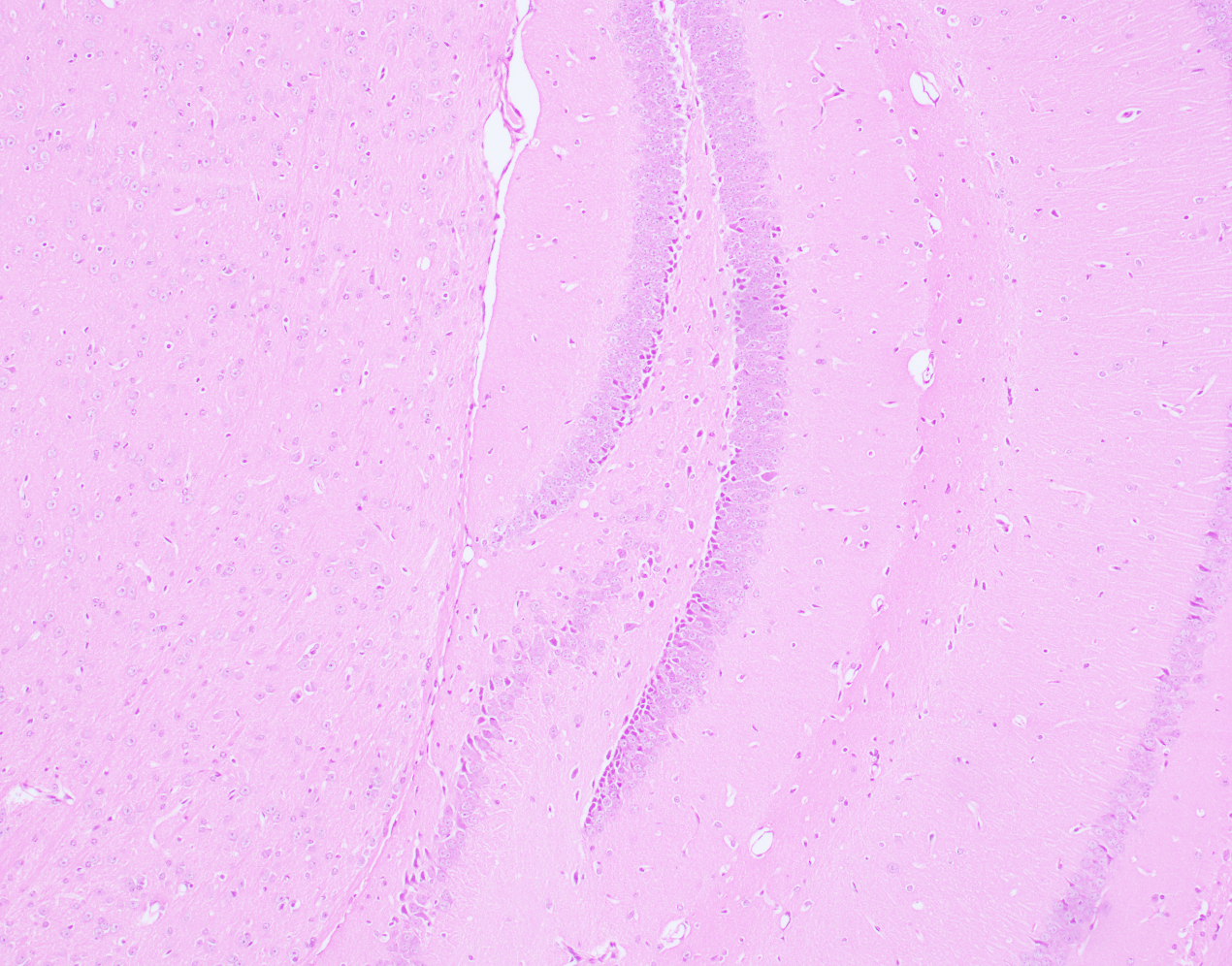

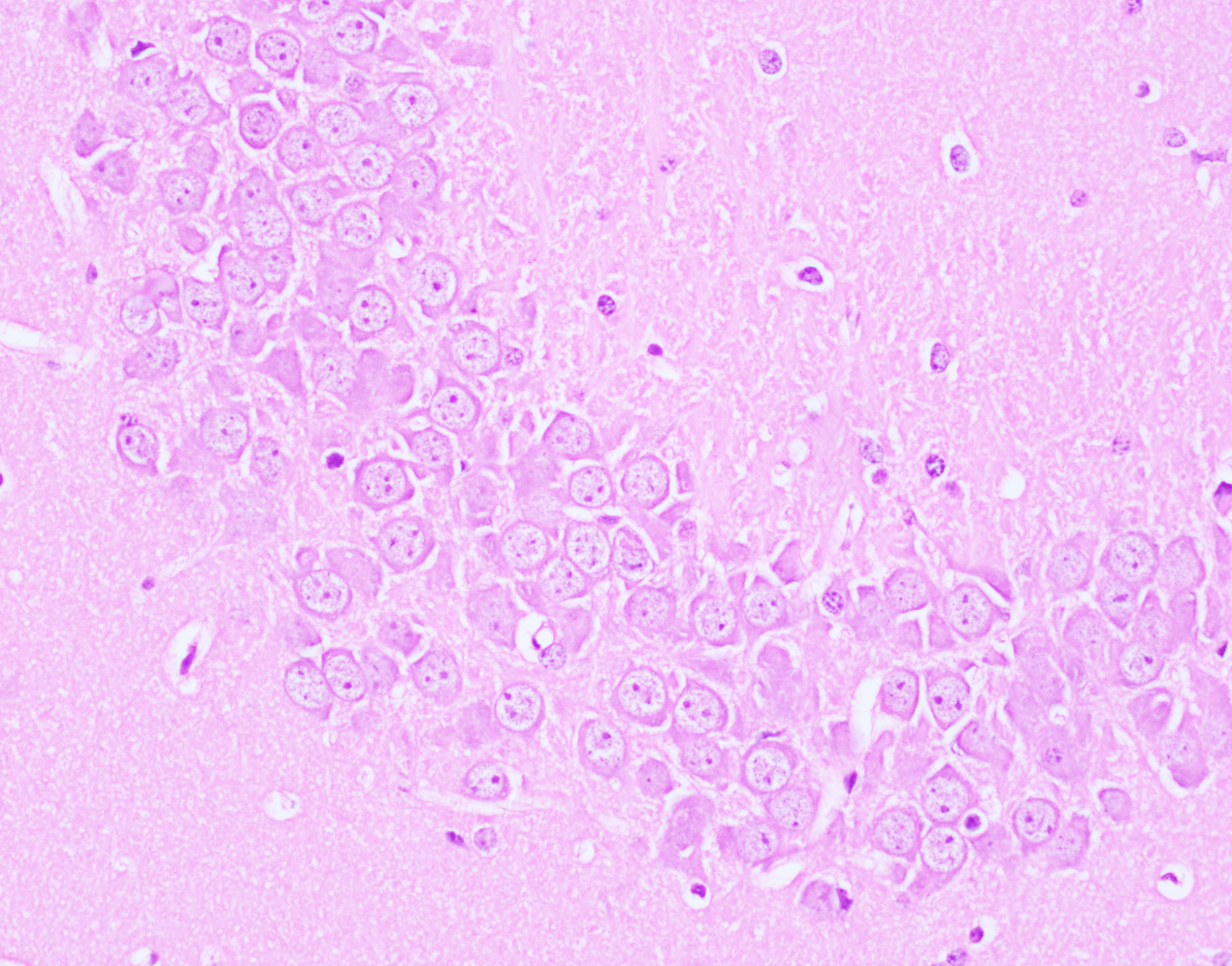

Supplement: Supplementary file 3 [file Table3.DOCX]

Immunohistochemistry


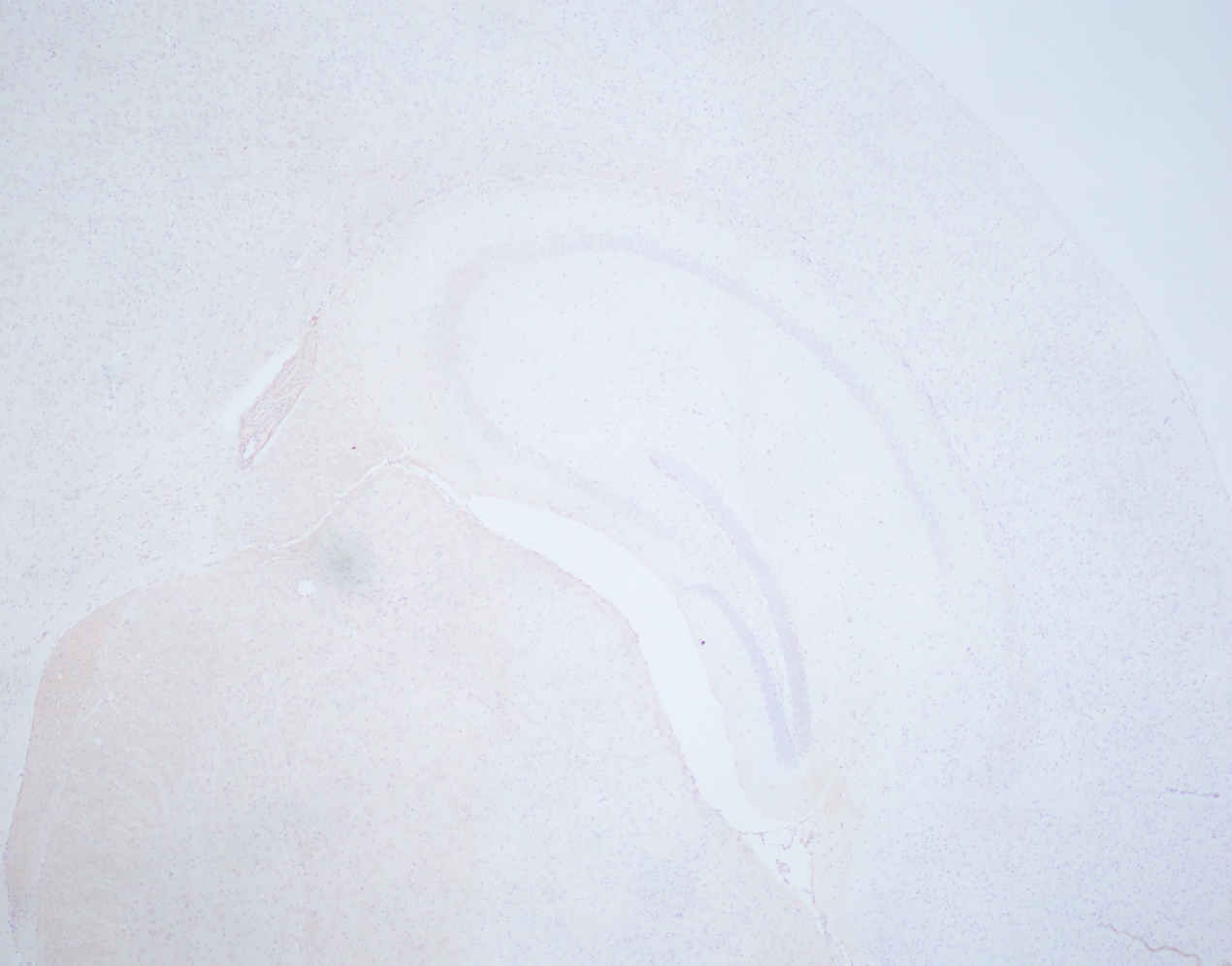

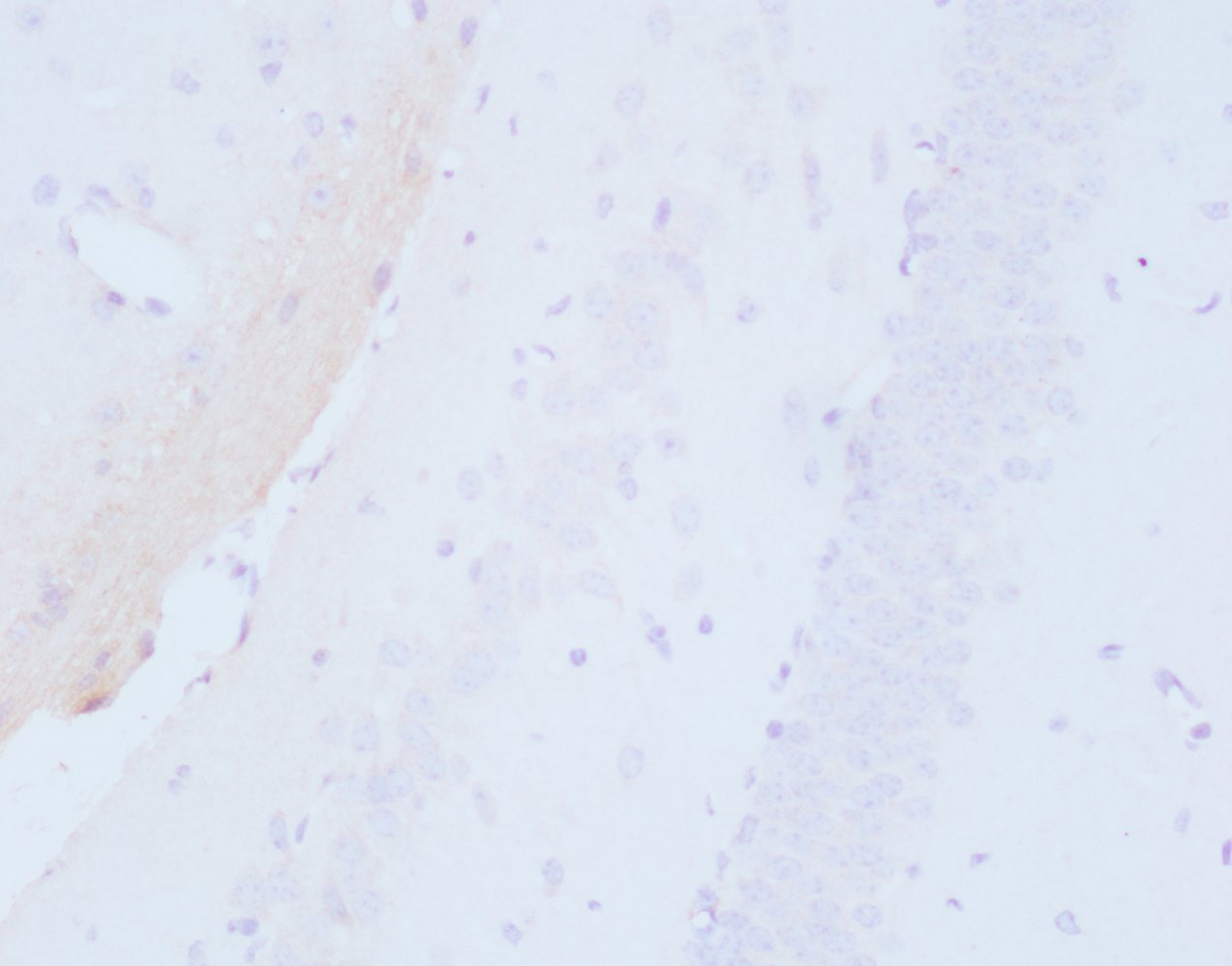

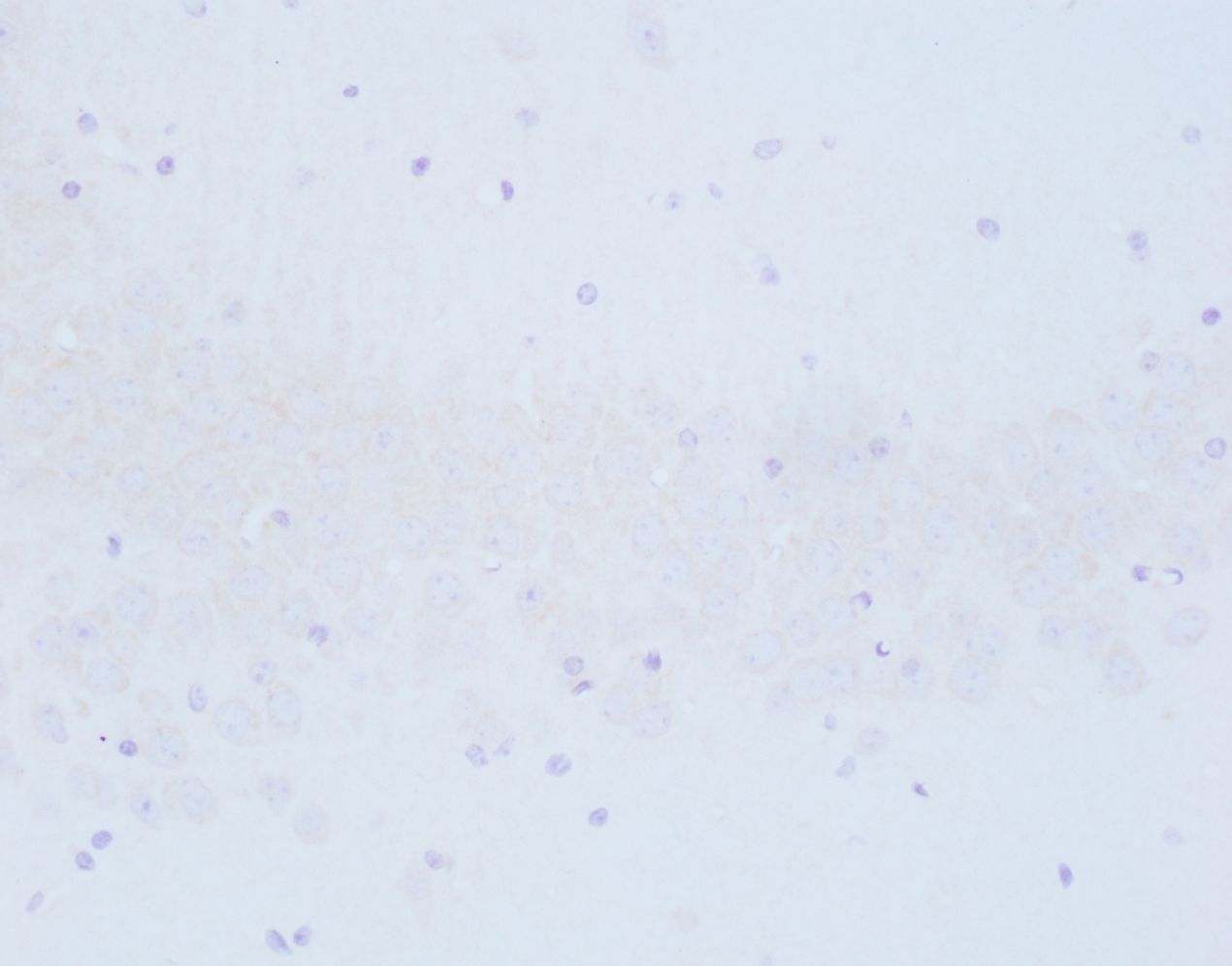

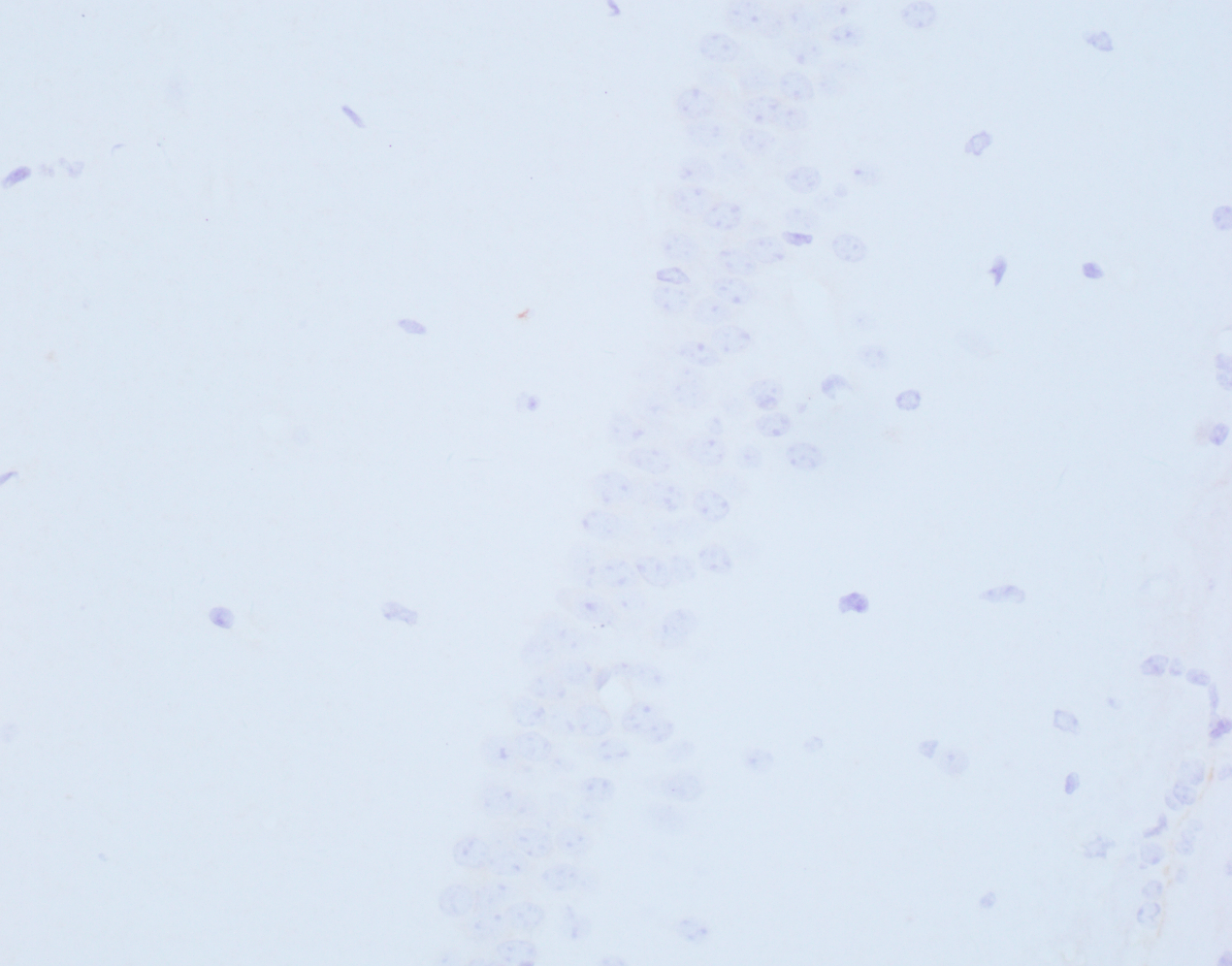

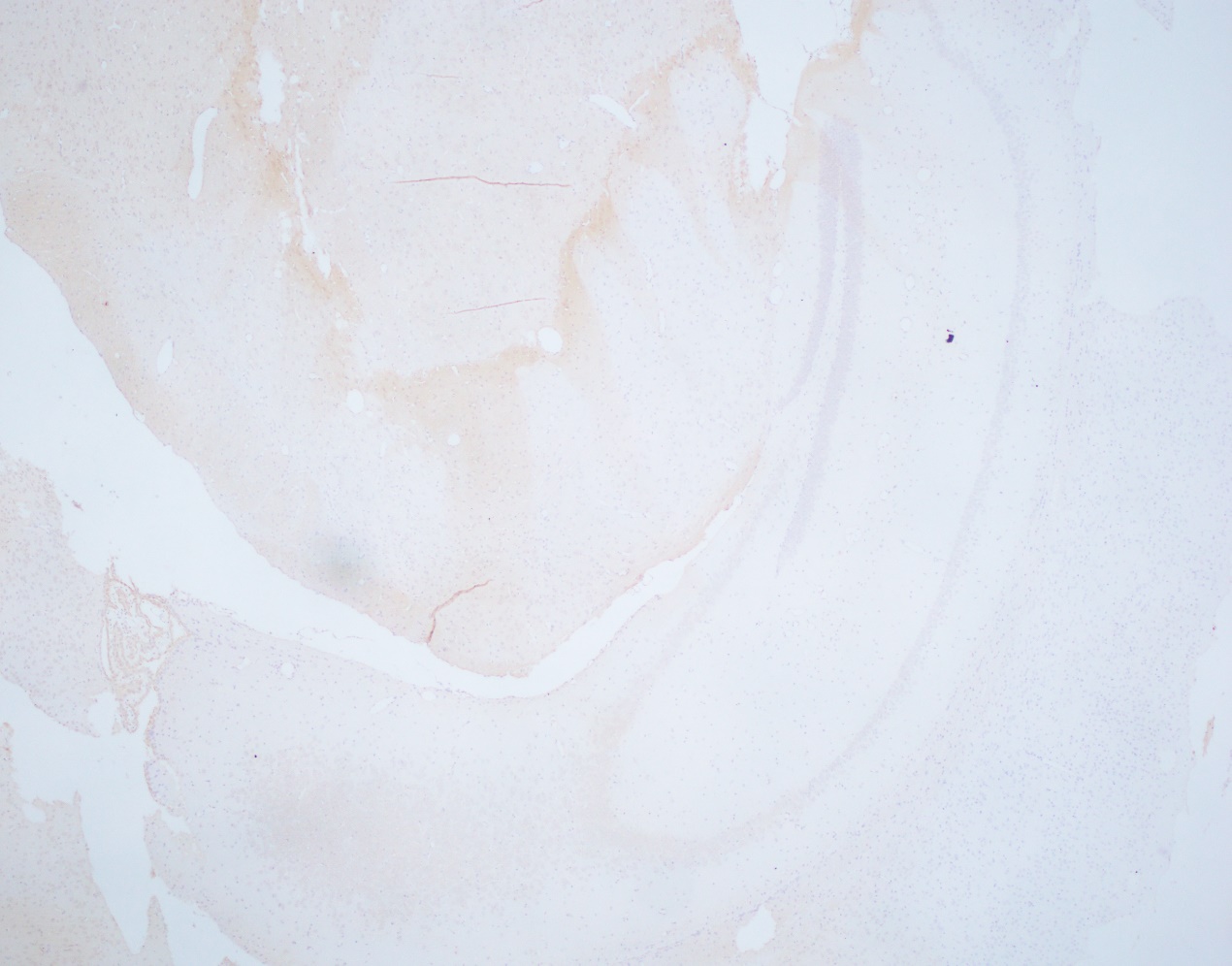

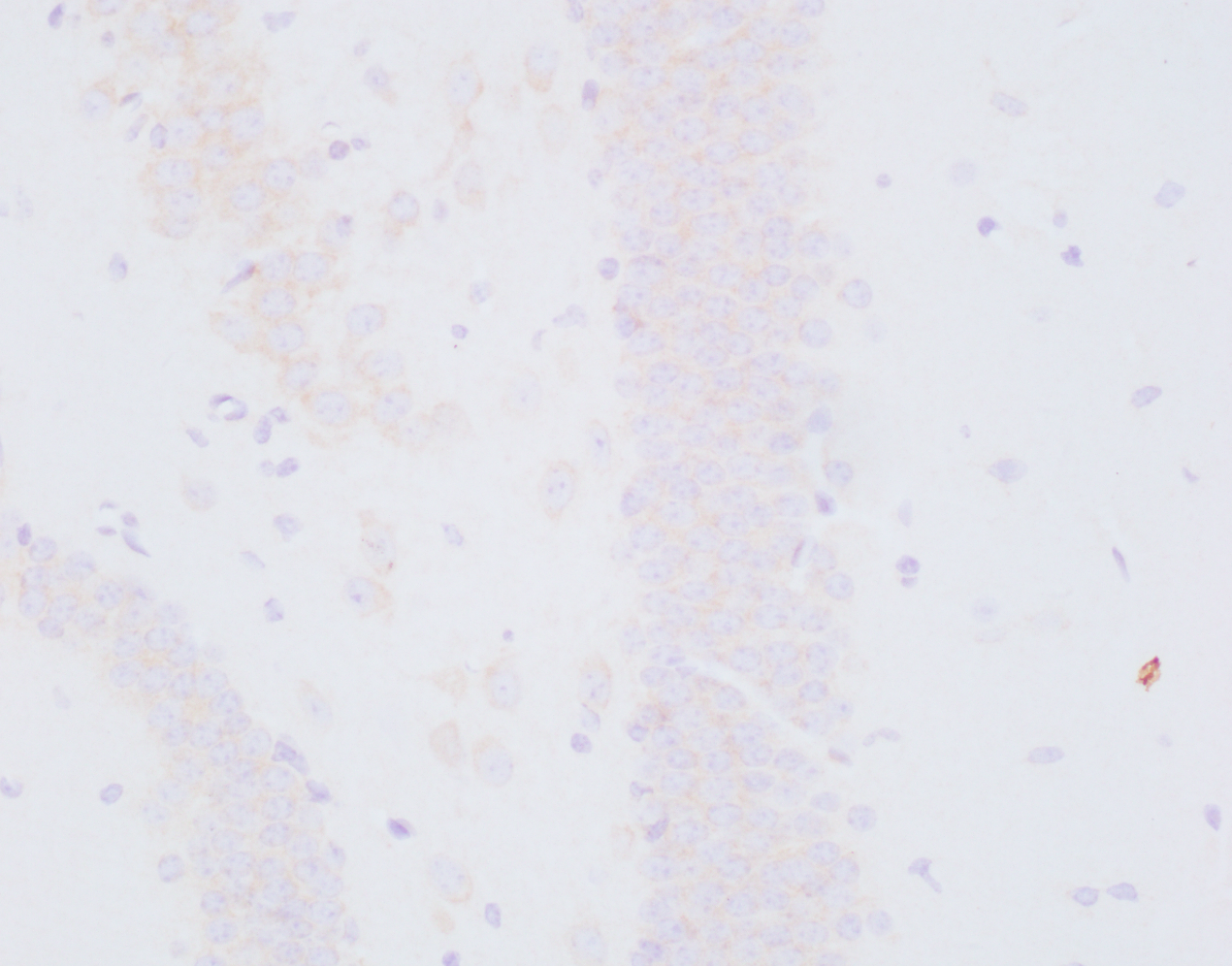

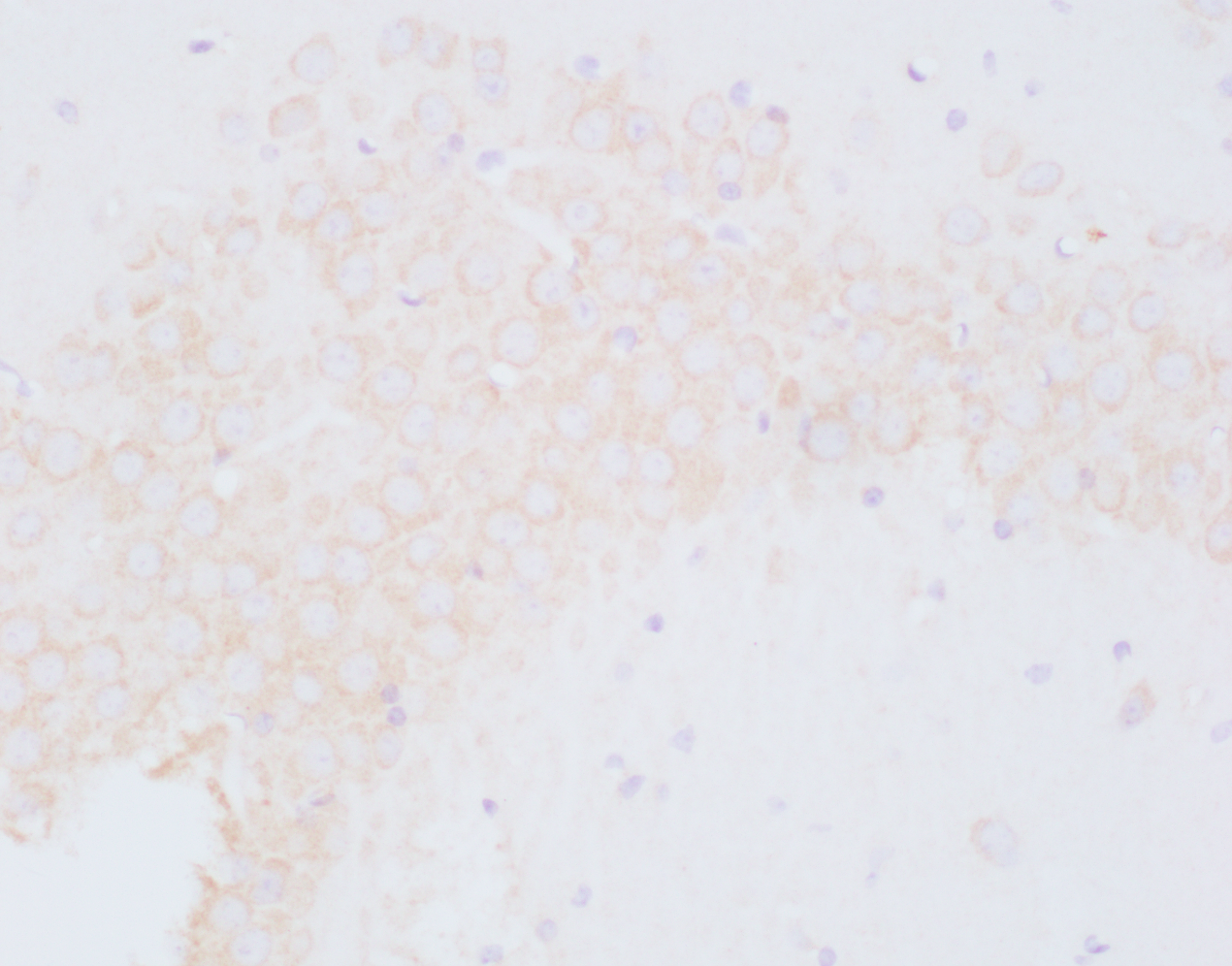

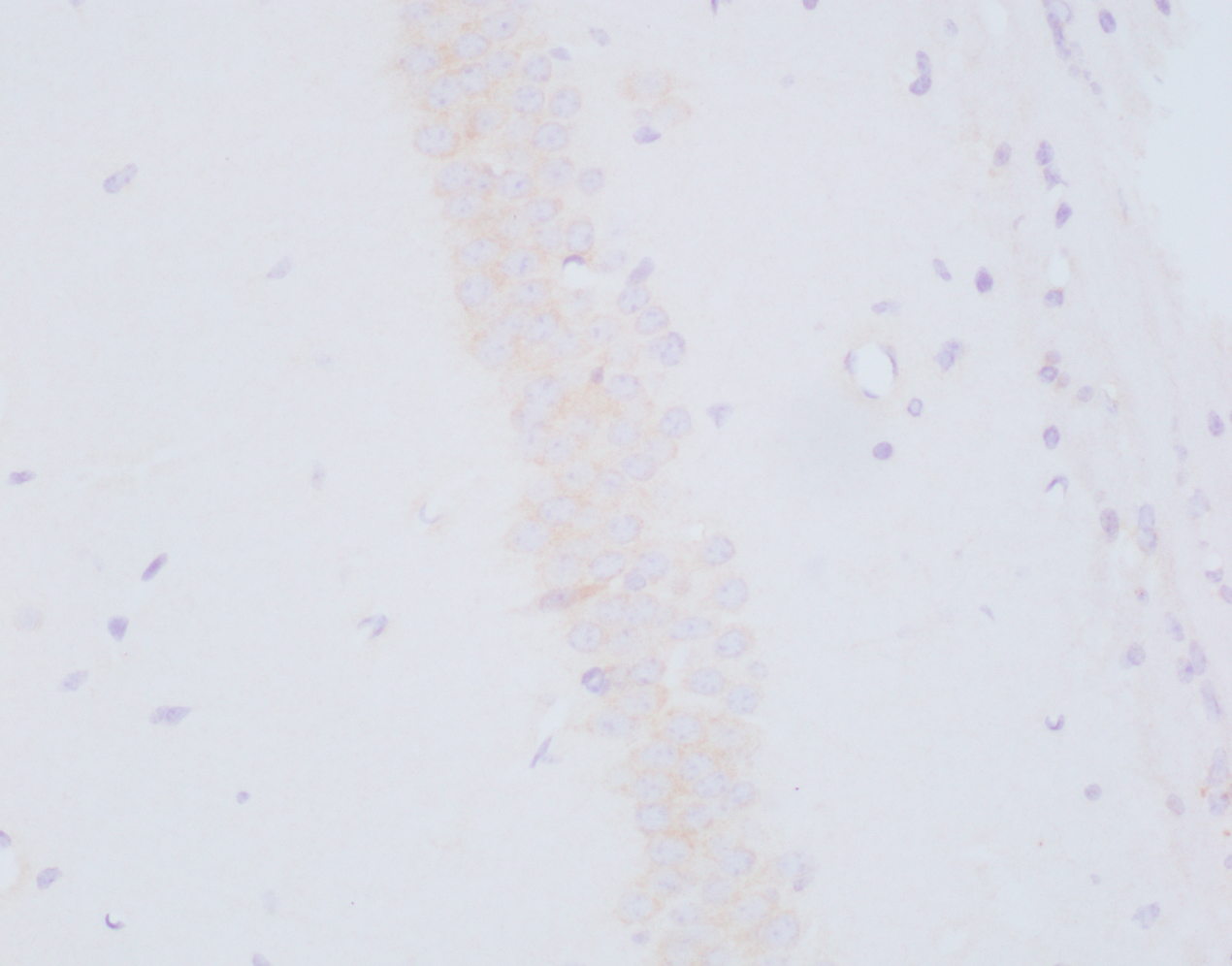

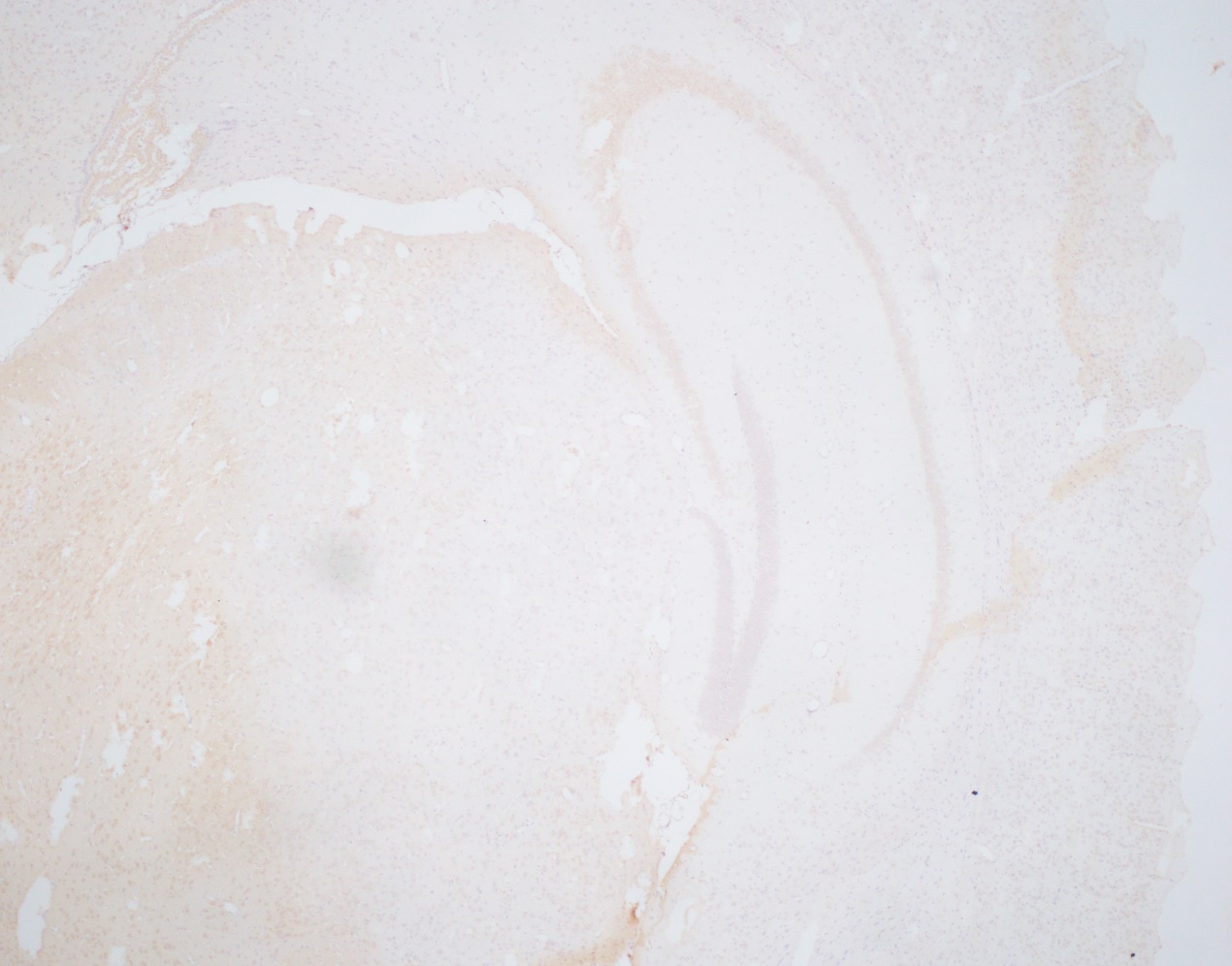

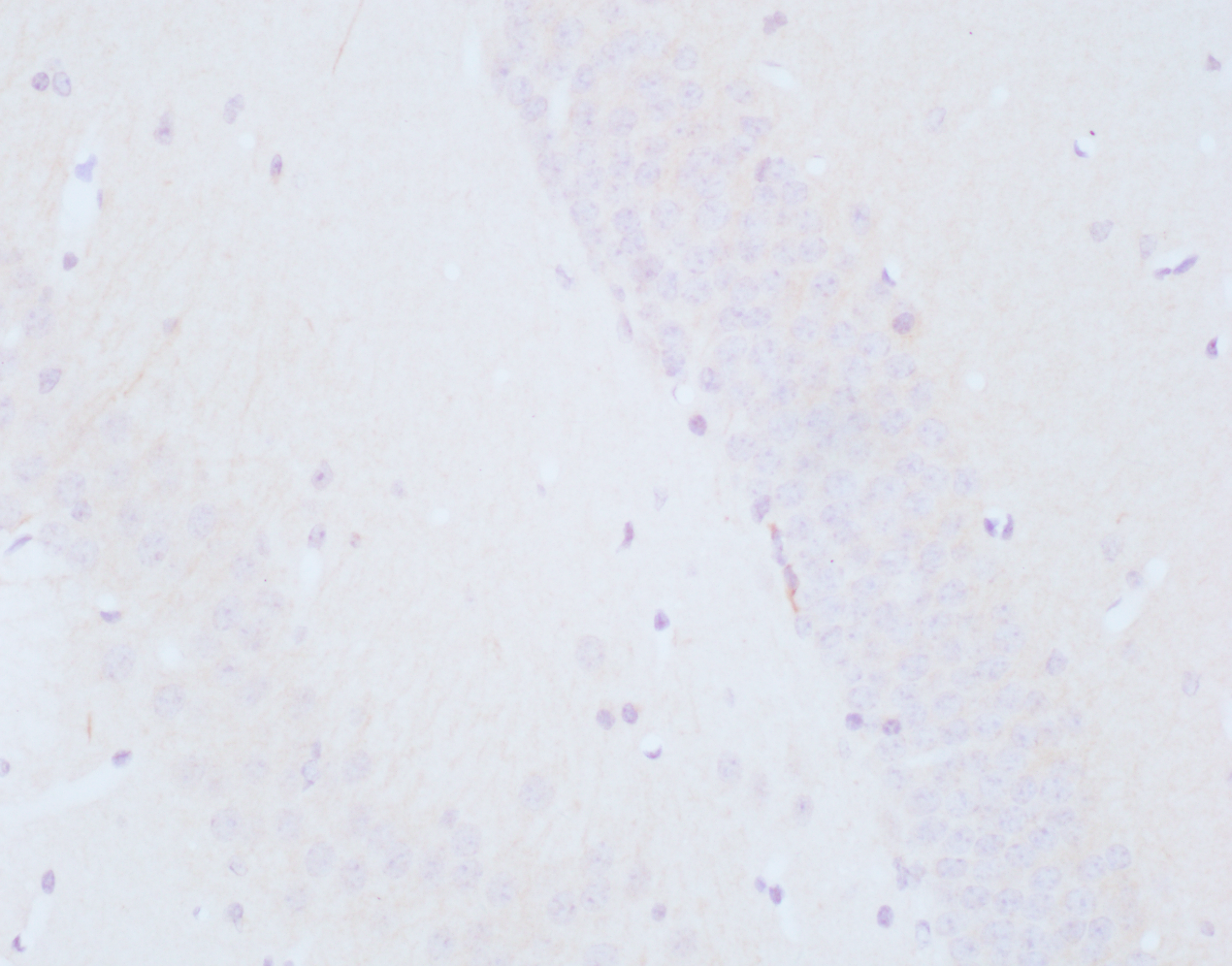

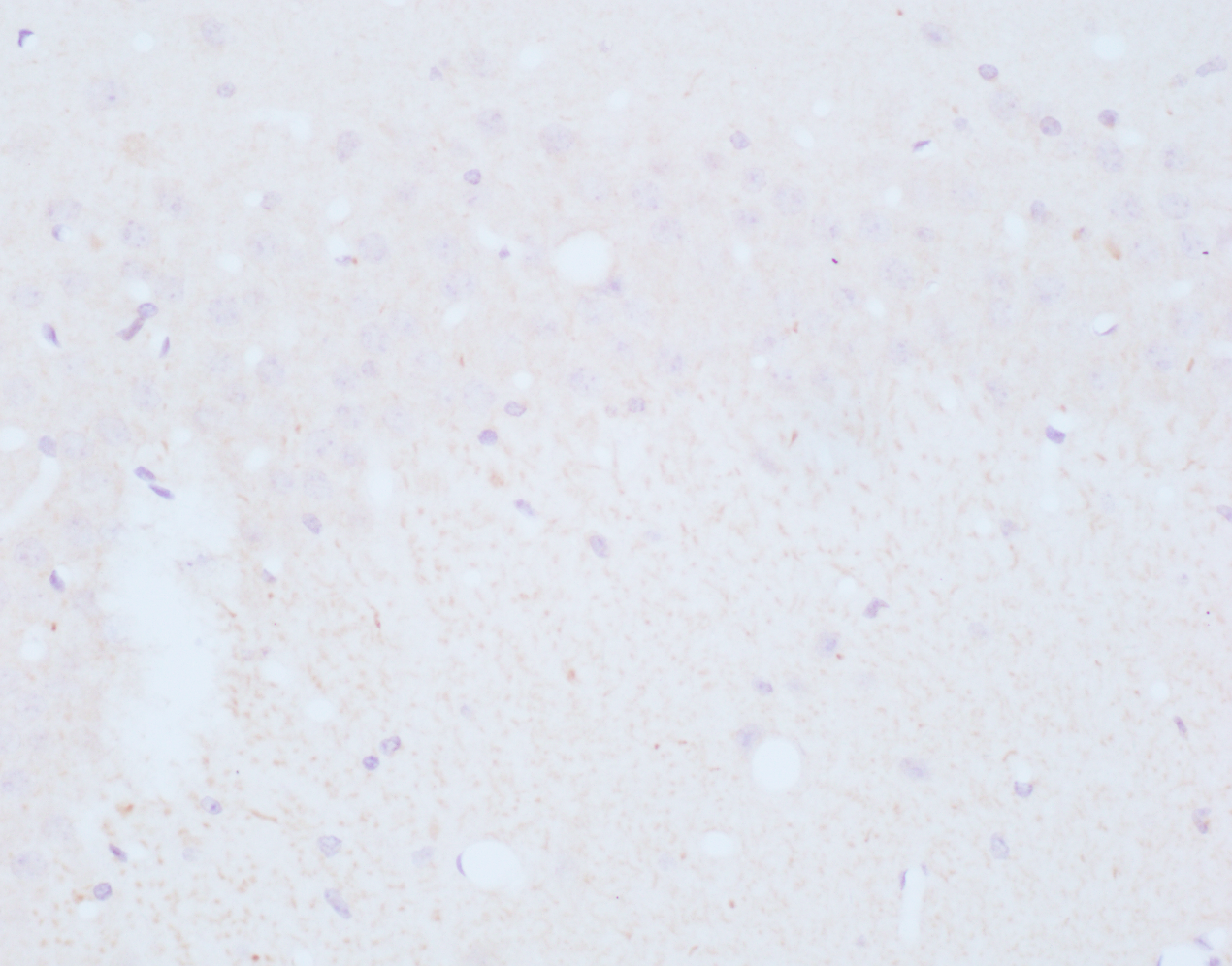

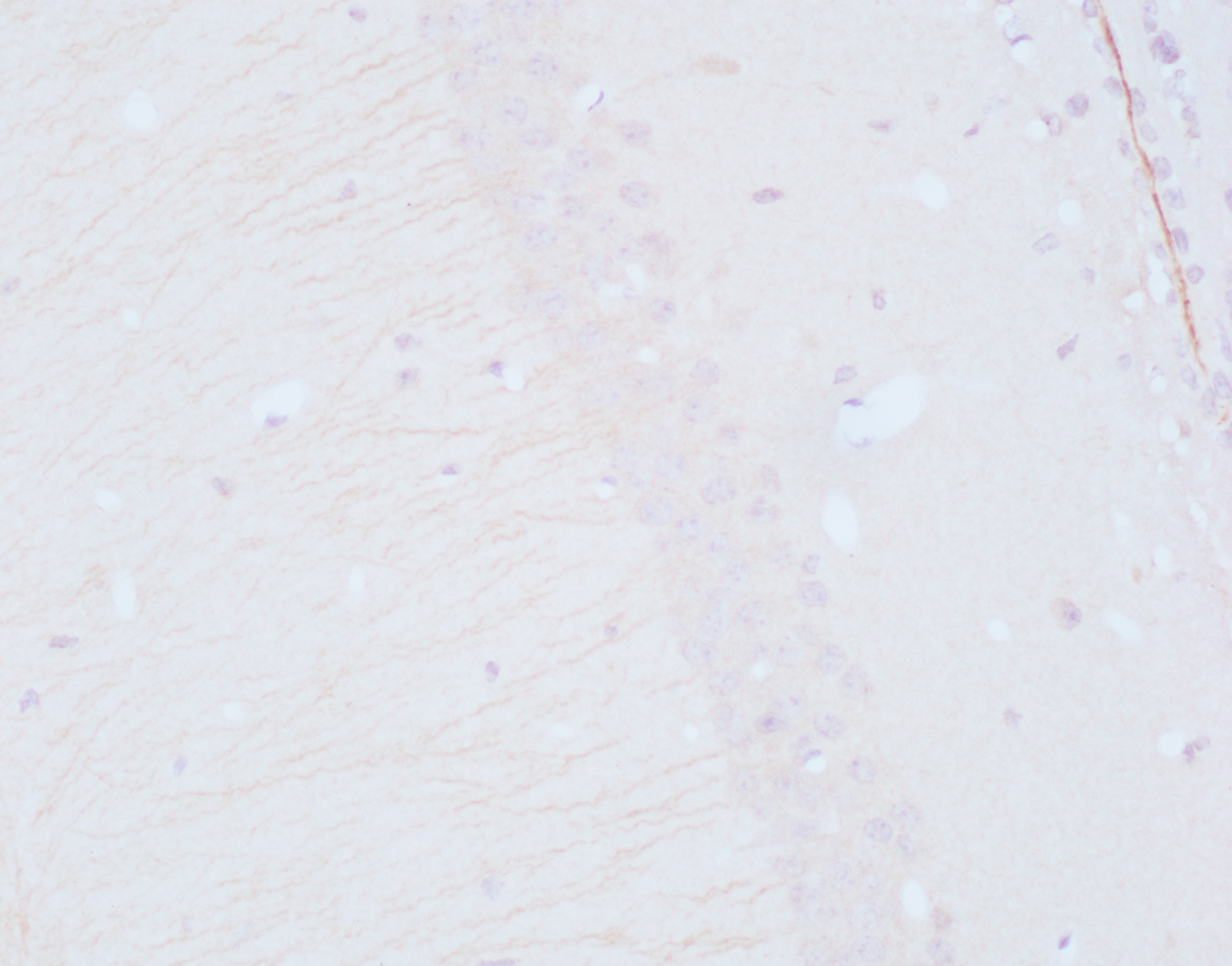

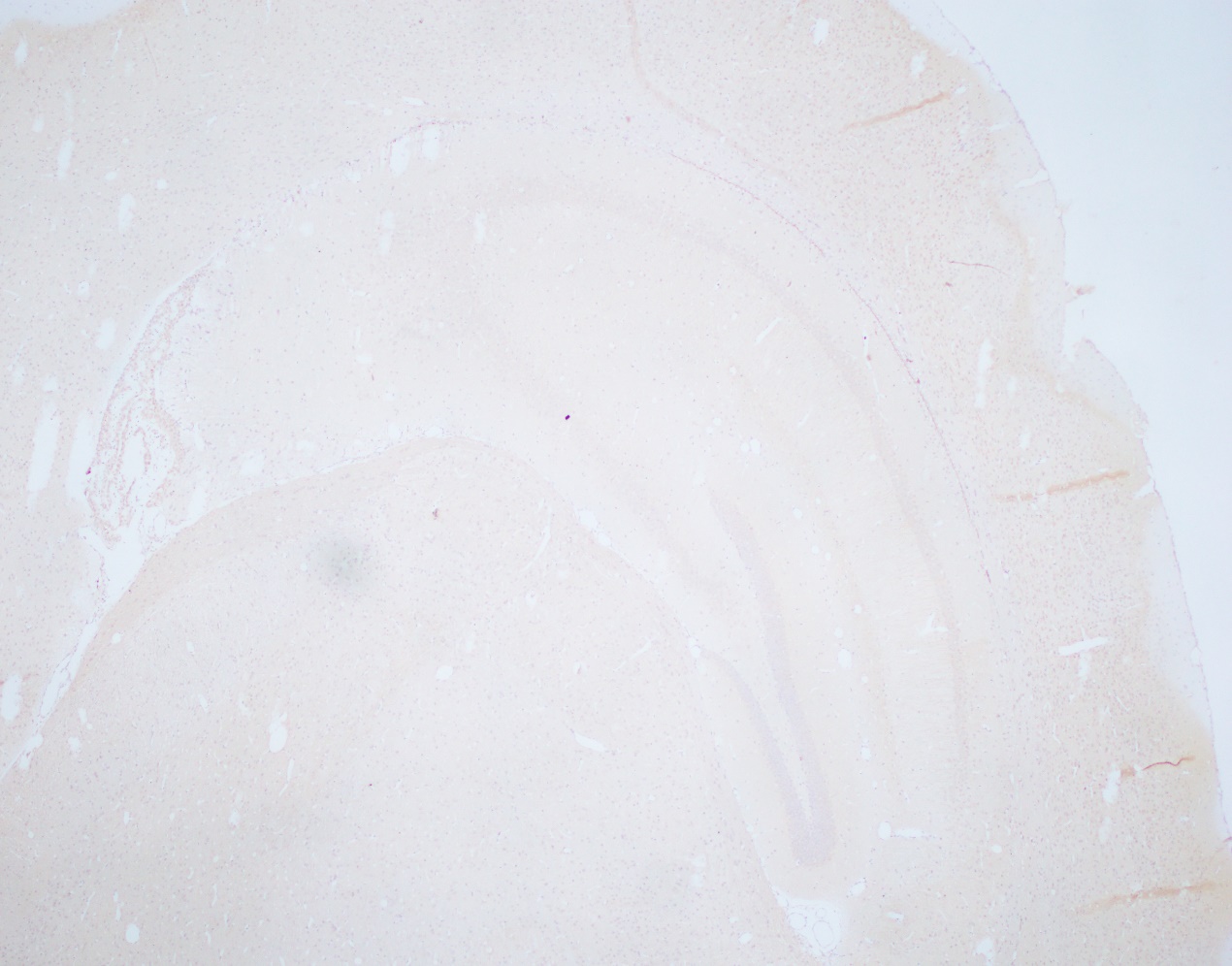

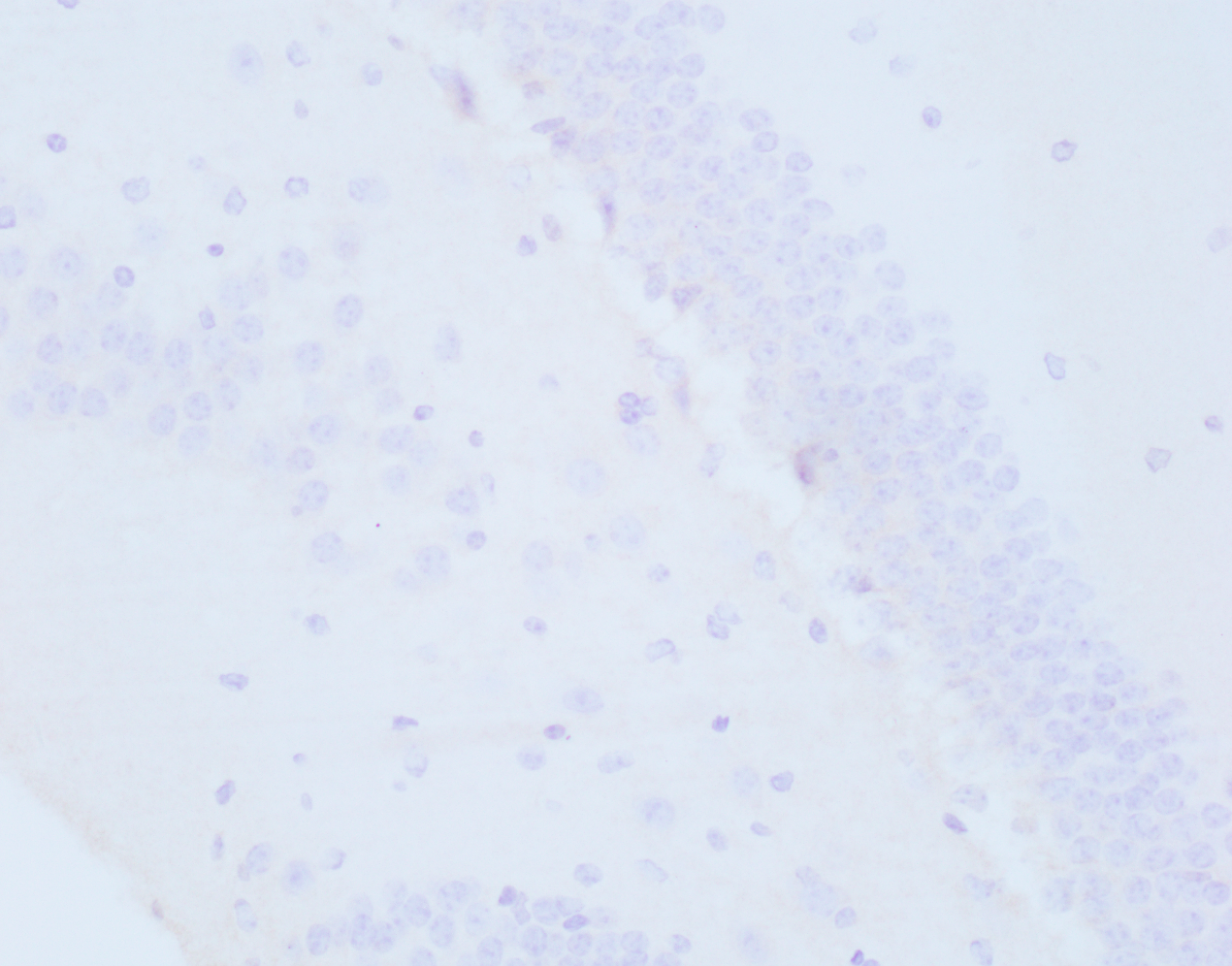

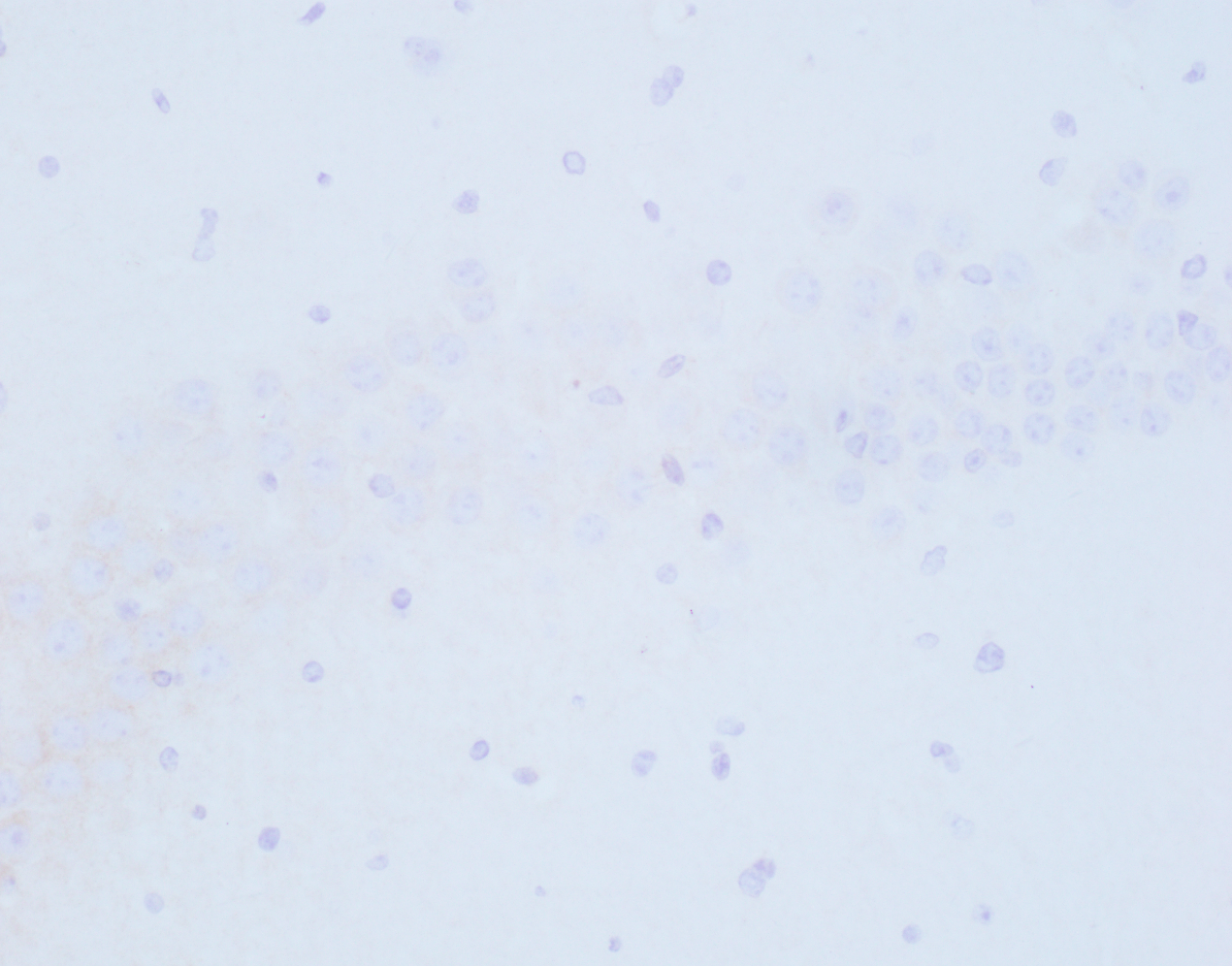

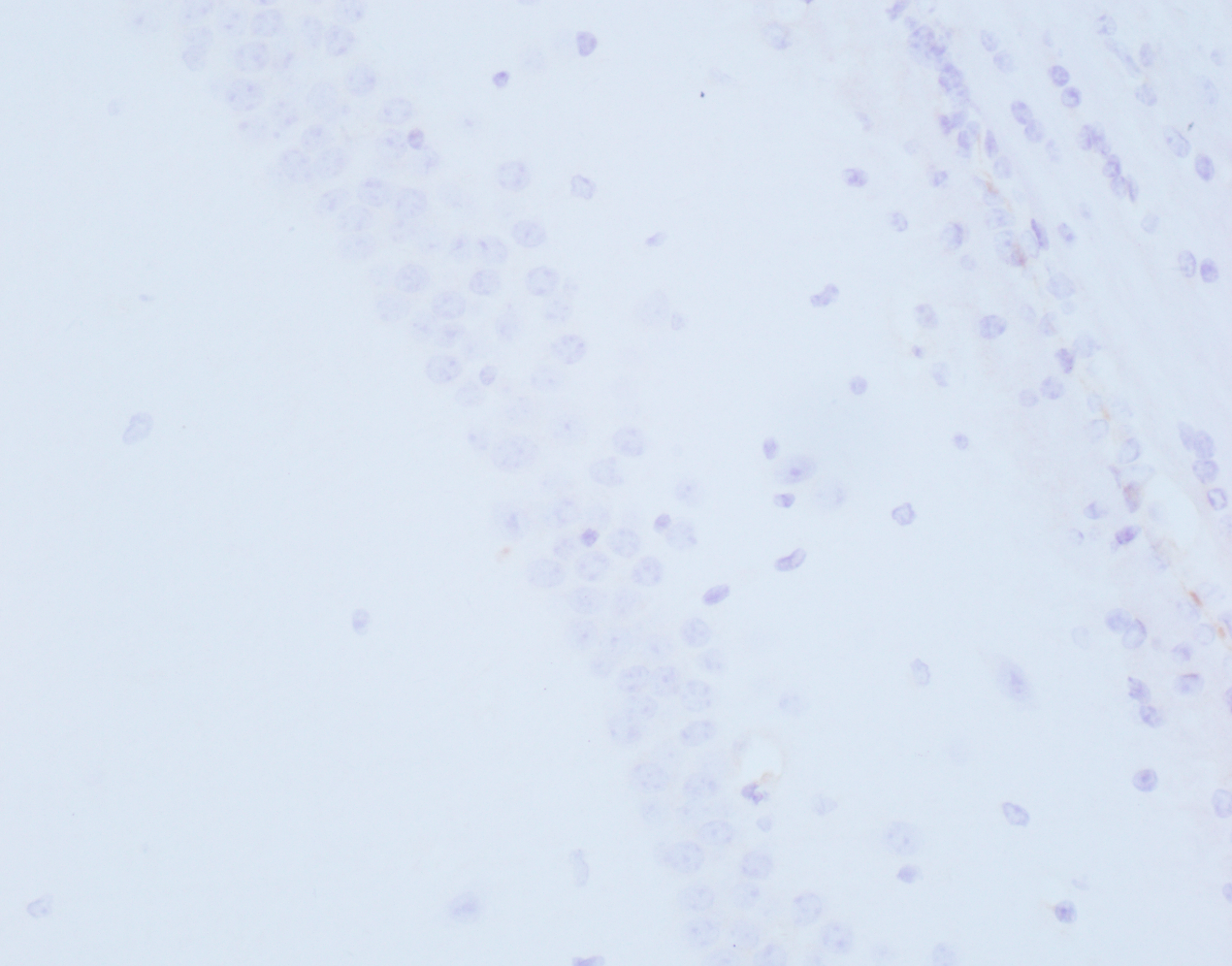

Supplement: Supplementary file 5 [file Table4.DOCX]
